# Transkingdom mechanism of MAMP generation by chitotriosidase feeds oligomeric chitin from fungal pathogens and allergens into TLR2-mediated innate immune sensing

**DOI:** 10.3389/fimmu.2025.1497174

**Published:** 2025-03-03

**Authors:** Tzu-Hsuan Chang, Yamel Cardona Gloria, Margareta J. Hellmann, Timmy Richardo, Carsten Leo Greve, Didier Le Roy, Thierry Roger, Francesca Bork, Stefanie Bugl, Johanna Jakob, Johannes Sonnberger, Lydia Kasper, Bernhard Hube, Stefan Pusch, Neil A. R. Gow, Morten Sørlie, Anne Tøndervik, Bruno M. Moerschbacher, Alexander N. R. Weber

**Affiliations:** ^1^ Department of Innate Immunity, Institute of Immunology, University of Tübingen, Tübingen, Germany; ^2^ Institute for Biology and Biotechnology of Plants, University of Münster, Münster, Germany; ^3^ Infectious Diseases Service, Department of Medicine, Lausanne University Hospital and University of Lausanne, Lausanne, Switzerland; ^4^ Department of Microbial Pathogenicity Mechanisms, Leibniz Institute for Natural Product Research and Infection Biology, Hans Knoell Institute, Jena, Germany; ^5^ Institute of Microbiology, Friedrich Schiller University, Jena, Germany; ^6^ Department of Neuropathology, Institute of Pathology, Ruprecht-Karls-University Heidelberg and German Cancer Consortium (DKTK), Clinical Cooperation Unit (CCU) Neuropathology, German Cancer Research Center (DKFZ), Heidelberg, Germany; ^7^ Department of Biosciences, Medical Research Council Centre for Medical Mycology at The University of Exeter, University of Exeter, Exeter, United Kingdom; ^8^ Department of Chemistry, Biotechnology, and Food Science, Norwegian University of Life Sciences, Ås, Norway; ^9^ Department of Biotechnology and Nanomedicine, Stiftelsen for Industriell og Teknisk Forskning (SINTEF) Industry, Trondheim, Norway; ^10^ iFIT – Cluster of Excellence (EXC 2180) “Image-Guided and Functionally Instructed Tumor Therapies”, University of Tübingen, Tübingen, Germany; ^11^ CMFI – Cluster of Excellence (EXC 2124) “Controlling Microbes to Fight Infection”, University of Tübingen, Tübingen, Germany; ^12^ Deutsches Konsortium für Translationale Krebsforschung (DKTK; German Cancer Consortium), Partner Site Tübingen, Department of Immunology, University of Tübingen, Tübingen, Germany

**Keywords:** chitin, chitotriosidase, N-acetyl-glucosamine, Toll-like receptor (TLR), inflammation, myeloid cell, innate immunity, *Candida albicans*

## Abstract

**Introduction:**

Chitin is a highly abundant polysaccharide in nature and is linked to immune recognition of fungal infections and asthma in humans. Ubiquitous in fungi and insects, chitin is absent inmammals and plants and, thus, represents a microbeassociatedmolecular pattern (MAMP). However, highly polymeric chitin is insoluble, which potentially hampers recognition by host immune sensors. In plants, secreted chitinases degrade polymeric chitin into diffusible oligomers, which are “fed to” innate immune receptors and co-receptors. In human and murine immune cells, a similar enzymatic activity was shown for human chitotriosidase (CHIT1), and oligomeric chitin is sensed via an innate immune receptor, Toll-like receptor (TLR) 2. However, a complete system of generating MAMPs from chitin and feeding them into a specific receptor/co-receptor-aided sensing mechanism has remained unknown in mammals.

**Methods:**

The effect of the secreted chitinolytic host enzyme, CHIT1, on the TLR2 activity of polymeric chitin preparations from shrimps, house dust mites and the fungal pathogen Candida albicans was assessed in vitro using cell lines and primary immune cells. Moreover, the regulation of CHIT1 was analyzed.

**Results:**

Here, we show that CHIT1 converts inert polymeric chitin into diffusible oligomers that can be sensed by TLR1/TLR2 co-receptor/receptor heterodimers, a process promoted by the lipopolysaccharide binding protein (LBP) and CD14. Furthermore, we observed that *Chit1* is induced via the b-glucan receptor Dectin-1 upon direct contact of immortalized human macrophages to the fungal pathogen *Candida albicans*, whereas the defined fungal secreted aspartyl proteases, Sap2 and Sap6, from *C. albicans* were able to degrade CHIT1 *in vitro*.

**Discussion:**

Our study shows the existence of an inducible system of MAMP generation in the human host that enables contact-independent immune activation by diffusible MAMP ligands with a striking similarity to the plant kingdom. Moreover, this study highlights CHIT1 as a potential therapeutic target for TLR2-mediated inflammatory processes that are fueled by oligomeric chitin.

## Introduction

Chitin, a hydrophobic polymer of β-1,4-linked *N*-acetylglucosamine (GlcNAc), is abundant in nature and can be found, e.g., in the cell wall of fungi, the exoskeletons of arthropods such as crustaceans and insects, and in nematodes (reviewed in 1–3). However, chitin does not exist in mammals and plants (2, 4, 5). In these organisms, chitin is a model microbe-associated molecular pattern (MAMP), as evidenced by the existence of chitin-mediated activation of immune responses through pattern recognition receptors (PRRs) (5, 6). Chitin is sensed through the receptor CERK1 and its co-receptor CEBiP in plants (7, 8) and in humans by Toll-like receptor (TLR) 2 on immune cells (9) and by FIBCD1 or LYSMD3 on epithelial cells (10, 11). During initial exposure, the mammalian host encounters chitin in a highly polymeric and insoluble form—e.g., the exoskeleton particles of house dust mites or the cell wall of pathogenic fungi—a form that has been considered immunologically inert (4). Meanwhile, chitin oligomers of 6 (DP6) and more GlcNAc subunits have emerged as potent immunostimulants sensed through PRRs in plants (7) and mammals (9). This begged two questions. First, how are oligomeric chitin subunits generated in the mammalian host? Second, how are the still rather poorly soluble and hydrophobic oligomers transferred to and sensed by PRRs?

The generation of MAMPs from complex, polymeric precursors has been observed, for example, in *Drosophila melanogaster* and plants. In *D. melanogaster*, Gram-negative bacteria-derived binding protein 1 (GNBP1) was reported to hydrolyze Gram-positive peptidoglycan to generate immune-active muropeptides (12), while in plants, secreted chitinases hydrolyze fungal cell walls to generate chitin oligomers sensed by membrane-expressed chitin receptor complexes (reviewed in 5). Many plant chitinases belong to the family of glycoside hydrolase 18 (GH18), a protein family that exists in plants, fungi, bacteria, actinomycetes, insects, and humans (13–15). In addition to acidic mammalian chitinase, humans produce chitotriosidase (CHIT1, also abbreviated as HCHT) (16). CHIT1 is expressed by neutrophils, macrophages, or epithelial cells (17–19) and the dominant chitinase in the human lung (20). CHIT1 overexpression in inflammatory conditions was first noted for Gaucher disease (MIM #230800), a lysosomal storage disease, where it serves as a therapy biomarker (21). CHIT1 is also elevated in patients with type 2 diabetes (22), amyotrophic lateral sclerosis (ALS) (20, 23–25), and childhood asthma (26), and it is generally accepted that CHIT1 reflects macrophage or microglial activation in these conditions. Although there is only minor alternative splicing (27), CHIT1 nevertheless exists in two major forms: a 50-kDa form mainly found in the blood and a 39-kDa form found predominantly in tissues (27, 28). Both forms contain a catalytic GH18 domain and display chitinolytic activity. Whereas the full-length, 50-kDa form of CHIT1 contains an additional C-terminal carbohydrate-binding module (CBM) domain, this domain is absent in the 39-kDa form, as it originates from posttranslational proteolytic cleavage of the C-terminus of the 50-kDa CHIT1 in lysosomes (27). Apart from a potential role in acting on other carbohydrates (even heparan sulfate and hyaluronic acid from the human host; 29), its ability to break down chitin has given rise to the notion that CHIT1 chitinase catalytic activity contributes to host defense against fungal infections especially targeting *Candida albicans* (18, 30–32). Conversely, another group observed a significant decrease in kidney fungal burden in *Chit1*-deficient mice compared to mice expressing the functional enzyme. They suggested that CHIT1-generated chitobiose (di-GlcNAc) acted as an immune suppressant (33). Likewise, in experimental *Klebsiella pneumoniae* lung infection, *Chit1* deficiency limited bacterial dissemination and improved survival, albeit via a different mechanism (34). Aside from fungal infections, in a murine model of interstitial lung disease, a manifestation of systemic sclerosis, *Chit1* deficiency prevented fibrotic lung damage, while transgenic *Chit1* overexpression promoted it (19). However, the lack of *Chit1* in an *in vivo* model of allergic lung inflammation showed reduced induction of regulatory T cells and hence a protective role (26). Depending on the context, CHIT1 thus seems to be able to promote or restrict immune responses. Its integration into the complex system of immunostimulation in the host and its putative role as a MAMP-generating enzyme are uncertain.

In addition to MAMP generation from polymeric and hydrophobic precursors, further activities may be required to optimize sensing by mammalian PRRs, such as transfer to and co-engagement of MAMPs by cell surface receptors. For example, the serum protein lipopolysaccharide (LPS) binding protein (LBP) and the soluble or membrane glycosylphosphatidylinositol (GPI)-anchored CD14 both contain hydrophobic binding pockets and shuttle LPS to plasma membrane TLR4 for initiation of signaling (35). Interestingly, LBP and CD14 also play a role in the transfer of mycobacterial lipopeptides to cell surface TLR2 (36, 37). TLR4 employs the soluble protein MD-2 as part of the actual receptor complex (35), while TLR2 is assisted by the transmembrane co-receptors TLR1 and TLR6 to sense tri- and di-acetylated mycobacterial lipopeptides, respectively (38, 39). In plants, the GPI-anchored CEBiP supports the chitin receptor CERK1 in chitin recognition at the protoplast (40). However, soluble accessory proteins or co-receptors able to shuttle oligomeric chitin to receptors (e.g., TLR2) have not been described in humans.

In this study, we examined the role of human CHIT1 in chitin sensing. We report that CHIT1 degrades polymeric, relatively inert chitin from *C. albicans* and house dust mite (HDM) to enable TLR2-dependent NF-κB activation and cytokine production. Interestingly, chitin sensing was not strictly dependent on direct contact of TLR2 to chitin but was rather mediated by diffusible oligomers that were generated in the presence of CHIT1. We further observed that chitin oligomers induced TLR1/TLR2 heterodimerization and that CD14 and LBP facilitated TLR2-NF-κB-dependent chitin sensing. Furthermore, we found that *Chit1* was induced via the β-glucan receptor Dectin-1 in murine macrophages exposed to pathogenic fungal cells, while secreted fungal proteases from *C. albicans* were able to degrade CHIT1 as a potential fungal mechanism of immune escape. Overall, our study unravels a novel and highly conserved system of chitin-based MAMP generation, shuttling, and recognition in mammals.

## Results

### CHIT1 generates diffusible TLR2-activating oligomeric chitin ligands

**Figure 1:**
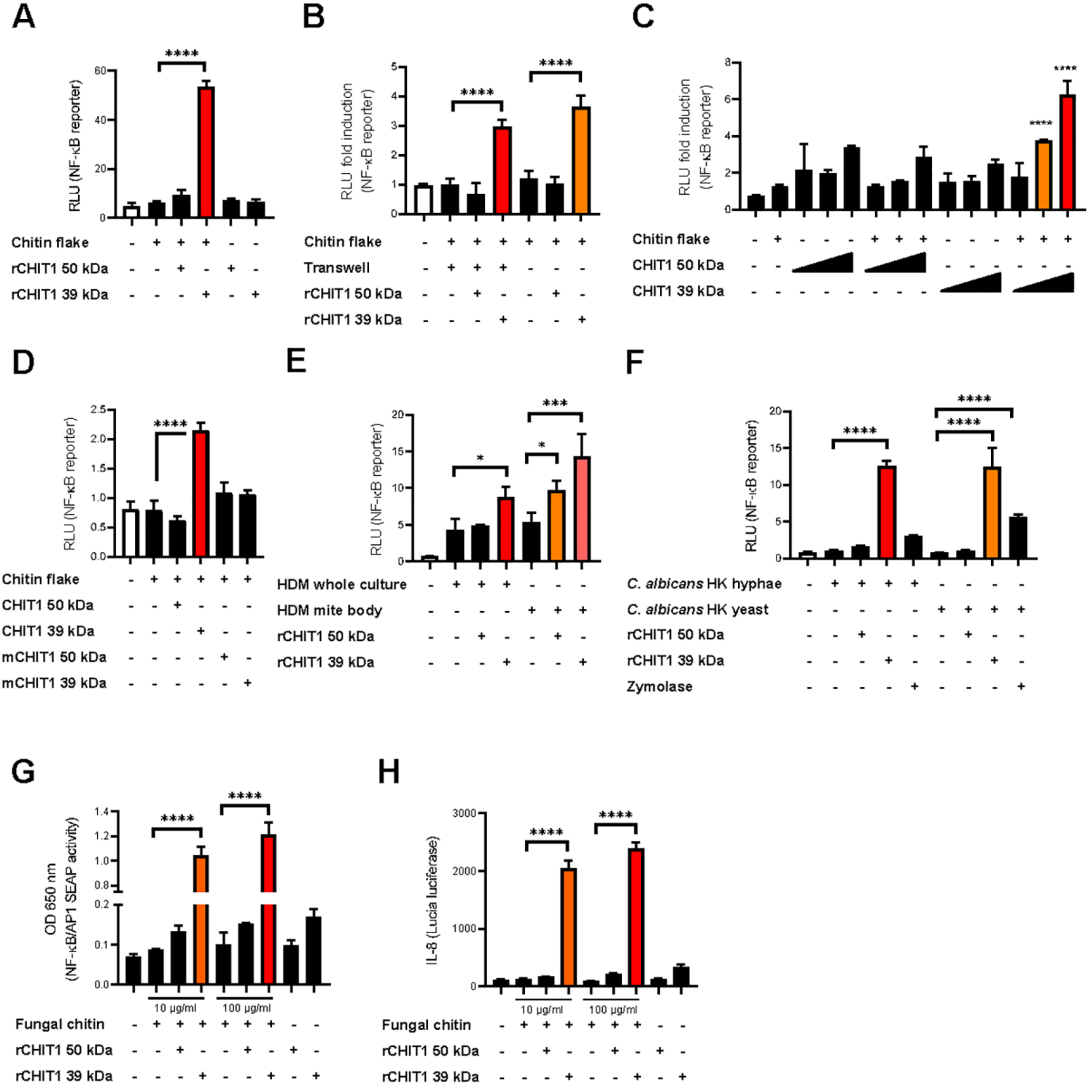
CHIT1 converts poorly immunogenic polymeric chitin into TLR2-active chitin. **(A–H)** CHIT1 digests of macroscopic chitin induce NF-κB activity in TLR2-overexpressing HEK293T cells. Recombinant CHIT1 isoforms [rCHIT1 in **(A, B, E–H)**] or supernatants from HEK293T cells expressing either wild-type CHIT1 **(C, D)** and/or catalytically inactive CHIT1 (mCHIT1) plasmids **(D)** were incubated with chitin flakes **(A–D)**, house dust mite (HDM) whole cultures or mite bodies **(E)**, *Candida albicans* hyphae and yeast forms **(F)**, or highly purified *C*. *albicans* fungal chitin **(G, H)**. After 18 h, the CHIT1-incubated culture supernatants were transferred to TLR2- and NF-κB-transfected HEK293T cells **(A–F)** or HEK-Dual™ hTLR2 reporter cells **(G, H)** for 18 h and lysates used for triplicate dual luciferase assays **(A–F)** (n = 3) or supernatants for triplicate NF-κB **(G)** or *IL8* **(H)** promotor reporter analysis. **(B)** The transwell setting was applied to avoid direct contact of undigested (−) and rCHIT1-digested (+) chitin flakes with the cells at the bottom of the plate. In **(A)** (n = 3), **(C)** (n = 3), **(D)** (n = 4), **(E)** (n = 2), **(F)** (n = 3), and **(G)** and **(H)** (n = 4 each), one representative of “n” biological replicates are shown (mean + SD for technical replicates). **(B)** (n = 2) represents combined data (mean + SD) from “n” biological replicates. *p < 0.05, ***p < 0.001, ****p < 0.0001 according to one-way ANOVA with Dunnett’s **(A)**, Tukey’s **(B, D, E)**, or Sidak’s **(C, F–H)** correction for multiple testing.

Based on the reported chitinase-based MAMP-generating system in plants (5), we first sought to investigate whether circulating enzymes that generate MAMPs for specific PRRs may play a role in chitin sensing in humans, e.g., for TLR2 activation (9). Since acidic mammalian chitinase displays exochitinase activity (41), it would be expected to release only di-GlcNAc subunits, which are too small to stimulate TLR2 (9). However, in contrast to acidic mammalian chitinase, CHIT1 is an endochitinase, cleaving inside chitin chains (17, 42). Thus, CHIT1 could enable the generation of longer oligomeric chitin fragments (31) that may directly stimulate TLR2. To test this hypothesis, purified and LPS-free commercially available macroscopic chitin flakes from shrimp (sieved size approximately 0.5 × 1–2 mm; **Supplementary Figure S1A** and Materials and Methods) were digested with purified recombinant 39-kDa (lacking the CBD) or 50-kDa (containing the CBD) recombinant CHIT1 [using expression constructs and purification procedures validated before (43); see Materials and Methods] and the product-containing reaction transferred to TLR2-expressing NF-κB reporter HEK293T cells (HEK293T-TLR2). Interestingly, while undigested macroscopic shrimp chitin was unable to activate TLR2, incubation with 39-kDa CHIT1 rendered it TLR2-active (**Figure 1A**). A similar emergence of TLR2 activity was observed (**Figure 1B**) when the experiment was repeated using a transwell setup (**Supplementary Figure S1B**, 8-µm pore size), separating the millimeter-scale chitin flakes and any fragments > 8 µm in the reaction from the cells. By doing so, we excluded the possibility that the observed CHIT1-induced TLR2-stimulating activity may be mediated by direct contact of cells (and hence TLR2) with the non-oligomeric macroscopic chitin. The acquisition of TLR2 activity of TLR2-transfected HEK293T cells (**Figure 1B**) was confirmed using Pam3 as a control stimulus (**Supplementary Figure S1C**). Conversely, lower-molecular-weight chitin oligomers (termed C10-15 throughout; estimated molecular weight 2,000–3,000, DP10-15, size ~5 nm; 9) were able to pass the filter pores and activate the cells (**Supplementary Figure S1C**). Although recombinant CHIT1 was clearly not able to activate TLR2 by itself, we further ruled out a possible role of confounding contaminants in recombinant CHIT1 protein preparations by ectopically expressing CHIT1 from a plasmid in HEK293T. In *CHIT1* plasmid-transfected cells, both CHIT1 isoform proteins were secreted as expected (see **Supplementary Figure S1D** for immunoblots of cell supernatants). Importantly, incubation of CHIT1-containing cell culture media from these cells with macroscopic chitin flakes also rendered the otherwise inactive macroscopic chitin TLR2-active upon transfer to TLR2–HEK293T cells (**Figure 1C**). To demonstrate further that activation by CHIT1 was truly due to enzymatic degradation of macroscopic chitin, we mutated the catalytic site of CHIT1 (D138A E140L, *cf.* 44; **Supplementary Figure S1E**). Medium from cells transfected with a mutant CHIT1 (mCHIT1) plasmid did contain secreted mCHIT1 (**Supplementary Figure S1D**) but, as expected, lacked chitinase activity (**Supplementary Figure S1F**). More importantly, the mutant was unable to generate TLR2 activity from macroscopic chitin flakes, compared to media from cells transfected with wild-type (WT) *CHIT1* plasmid (**Figure 1D**). We conclude that especially the 39-kDa form of CHIT1 is able to generate diffusible TLR2 activating ligands from macroscopic shrimp chitin. When analyzing digestion reactions from CHIT1 incubation with chitin flakes, we were able to detect highly acetylated products of DP2-4 (**Supplementary Figure S1G**). Such oligomers are too short to activate TLR2 (9), but the analysis verifies CHIT1 endochitinase activity. Generally, it is highly challenging to detect intermediate enzyme products of DP6 or greater from such a cell culture system as their half-life, concentration, solubility, and ionization efficiency during mass spectrometry are exceedingly low. Provided that Kimura et al. (45), albeit in a cell-free system (45), detected fully acetylated chitin oligomers of DP6 to be released from a similar macroscopic substrate, colloidal chitin, and that at least DP4 oligomers were confirmed, we consider it highly plausible that CHIT1 degradation releases intermediate products that are sufficiently long and abundant for TLR2 engagement, which occurs with nanomolar K_D_ (9).

We then went on to investigate whether this effect applied to polymeric chitin from other sources, such as the disease-relevant HDM, *Dermatophagoides pteronyssinus*, and the pathogenic fungus, *C. albicans*. Using the same digestion assay, we observed a significant increase of reporter activity in HEK293T-TLR2 cells, especially upon pre-incubation of *D. pteronyssinus* whole cultures and mite bodies with recombinant 39-kDa CHIT1 in both the normal and transwell setups (**Figure 1E**). The same effect was observed for chitin derived from heat-killed *C. albicans* yeast and hyphae (**Figure 1F**). In this experiment, zymolyase, an enzyme cleaving β-glucan chains, only had a minor effect that may be attributable to degrading the scaffolding with which chitin is integrated into the fungal cell wall (46). The experiment was also performed specifically with macromolecular, highly purified *C. albicans* chitin (47), which triggered drastically increased TLR2-mediated activity upon 39-kDa CHIT1 digestion (**Figures 1G, H**). Collectively, these data show that CHIT1 is able to generate potent and diffusible TLR2-activating MAMPs from polymeric, poorly activating macroscopic chitin, reminiscent of observations from *Drosophila* (12) and plants (5).

### CHIT1 binds to the fungal cell wall

**Figure 2:**
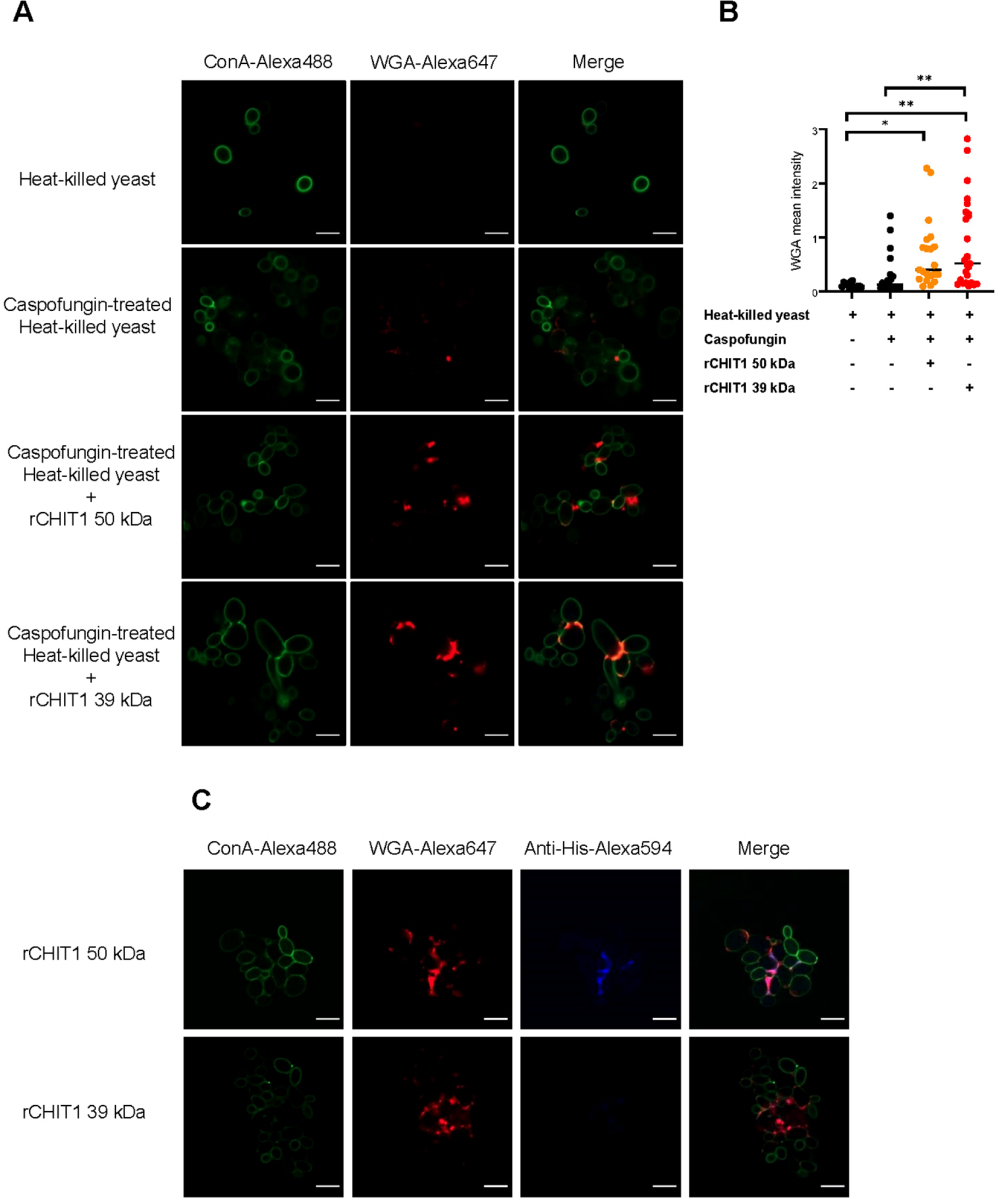
CHIT1 digestion increases chitin exposure in *Candida albicans* yeast. **(A, C)** Heat-killed *C*. *albicans* yeast cells were left untreated or treated with caspofungin (0.032 μg/mL) and CHIT1 as indicated. Cells were then stained with Alexa448-ConA (green) and Alexa647-WGA (red). Shown are representative single focal images of *C*. *albicans* yeast cells containing at least 5–20 yeast cells, with multiple images taken per condition across multiple biological replicates. **(B)** WGA fluorescent intensity was quantified, thresholds were applied to the ConA and WGA signals, and the intensity was measured using the Otsu method. The mean intensity of WGA signal was normalized to that of ConA. **(C)** To stain CHIT1 on caspofungin-treated, heat-killed *C*. *albicans* yeast, Alexa594-conjugated anti-His antibodies were used to detect the His-tag on CHIT1. In **(A)** (n = 3) and **(C)** (n = 2), one representative of “n” biological replicates is shown. **(B)** (n = 2) represents combined data (mean and individual points) from “n” biological replicates (each dot represents one quantified image). *p < 0.05, **p < 0.01 according to one-way ANOVA with Tukey’s correction for multiple testing. WGA, wheat germ agglutinin; ConA, concanavalin A.

To verify the effect of CHIT1 on fungal cell wall chitin, confocal fluorescence microscopy analysis of *C. albicans* yeast cells was performed upon incubation with CHIT1. For comparison, yeast cells were also treated with caspofungin, an inhibitor of β-1,3-d-glucan synthesis, which is known to increase chitin exposure on the fungal cell wall (48). Untreated and caspofungin-treated cells were heat-inactivated and pre-incubated with 39-kDa or 50-kDa CHIT1. Finally, yeast cells were labeled with Alexa647-conjugated, chitin-binding wheat germ agglutinin (WGA) lectin and Alexa488-conjugated concanavalin (Con) A, which binds to α-mannans and hence served as a control that should be unaffected by CHIT1. Confocal imaging revealed that WGA bound more prominently to *C. albicans* yeast cells after CHIT1 digestion (**Figure 2A**, quantified in **Figure 2B**), whereas the binding of ConA remained largely unchanged. Additionally, we stained for CHIT1 itself using anti-His primary and matching Alexa594-conjugated secondary antibodies. At the fungal cell wall, 50-kDa CHIT1 was visualized (**Figure 2C**), indicating a stable binding consistent with the presence of a CBD. No detectable binding of 39-kDa CHIT1 was observed, probably due to the lack of a CBD and hence only transient interaction with chitin (43). The lower retention time of the 39-kDa form of CHIT1 at the cell wall would be consistent with the generation of longer soluble oligomers, which are more favorable TLR2 ligands (9) due to lower processivity. In summary, recombinant CHIT1 can directly engage with the fungal cell wall, consistent with the release of oligomeric chitin fragments.

### Chitin binding involves multiple PRRs, whereas TLR2 can sense diffusible oligomeric chitin

**Figure 3:**
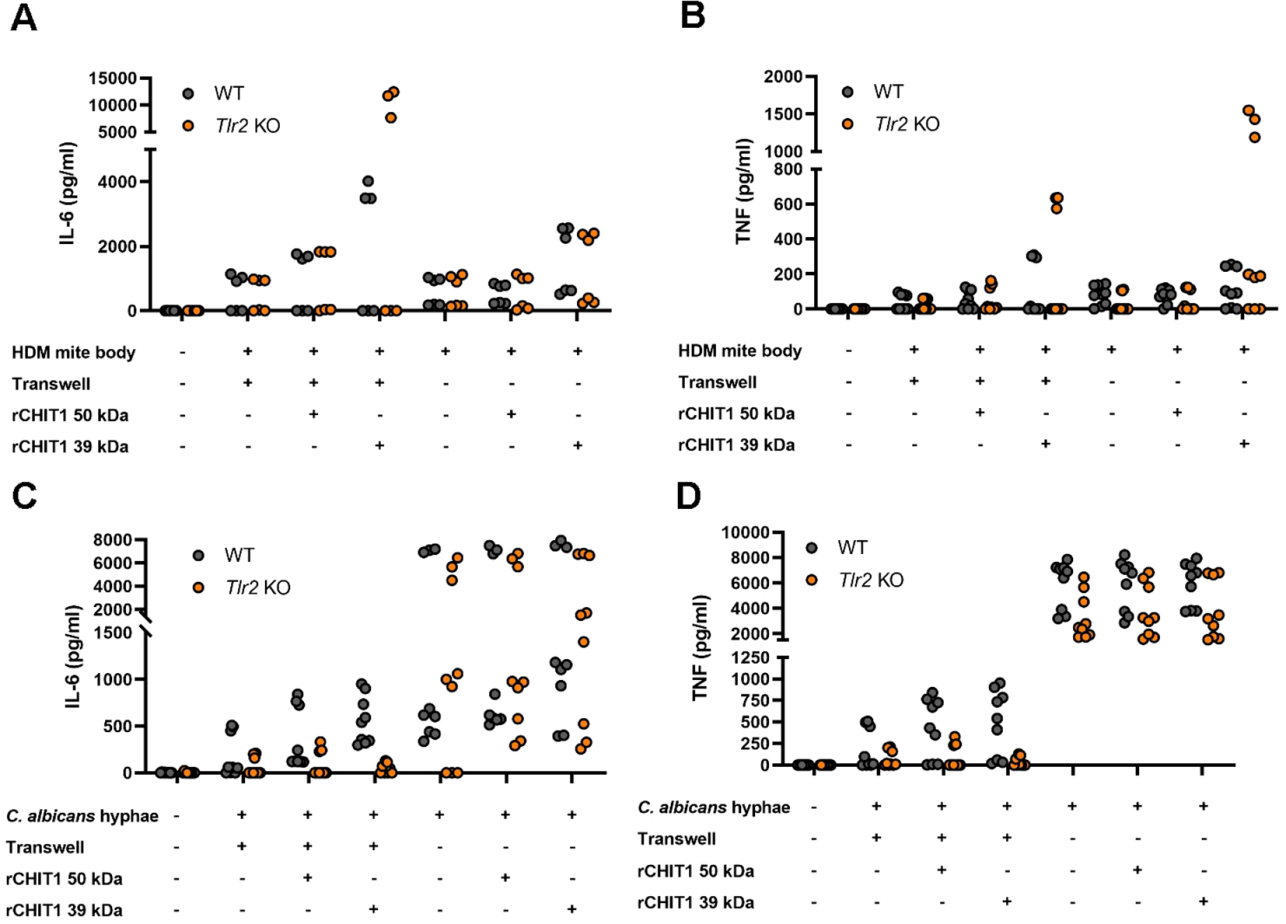
Diffusible chitin oligomers released from chitin-rich organisms elicit the secretion of TLR2-dependent pro-inflammatory cytokines in murine macrophages. **(A–D)** IL-6 and TNF secretion by WT or *Tlr2* KO murine BMDMs upon stimulation with the indicated organisms left undigested (−) or digested with either isoform of recombinant CHIT1 (+) either with transwell or direct addition, as assessed via ELISA. **(A–D)** n = 3 biological repeats with technical triplicates each are shown. WT, wild type; KO, knockout; BMDMs, bone marrow-derived macrophages.

The TLR2–HEK293T system lacks many PRRs except TLR2 and TLR2 co-receptors (49). To extend our insights to a more physiologically relevant system, primary bone marrow-derived macrophages (BMDMs) were stimulated with native and CHIT1-digested *D. pteronyssinus* HDMs (whole cultures and mite bodies) or *C. albicans* hyphae and yeast. BMDMs produced IL-6 and TNF in response to undigested *D. pteronyssinus* and *C. albicans*. However, the stimulatory capacity of HDM bodies (**Figures 3A, B**) and *C. albicans* hyphae (**Figures 3C, D**) increased following CHIT1 39-kDa digestion. Parallel analysis of *Tlr2* knockout (KO) BMDMs and use of the transwell setup showed that HDM preparations were not fully TLR2-dependent and thus probably contained other MAMPs that were not detectable in HEK293T cells but were increased by CHIT1 incubation and dominated over chitin in BMDMs. Moreover, BMDM levels of cytokine release were more similar when comparing transwell and direct incubation (**Figures 3A, B**). For *C. albicans* hyphae, however, the transwell setting clearly was associated with lower cytokine release but stronger TLR2 dependence than for direct contact (**Figures 3C, D**). The expected inter-individual differences in maximal release between primary cells from different animals precluded confirming these differences statistically. Nevertheless, observed trends suggest that in BMDMs and for *C. albicans*, TLR2 is able to elicit cytokine release to chitin-containing pathogens upon direct binding but, more importantly, can also sense diffusible ligands generated by CHIT1 for IL-6 and TNF secretion. Thus, CHIT1 digestion enables PRR-expressing cells to overcome the requirement of direct physical contact with chitin-bearing pathogens using oligomer- and diffusion-mediated distal chitin sensing.

### Oligomeric chitin signals via LBP, CD14, and TLR1/TLR2 heterodimers

**Figure 4:**
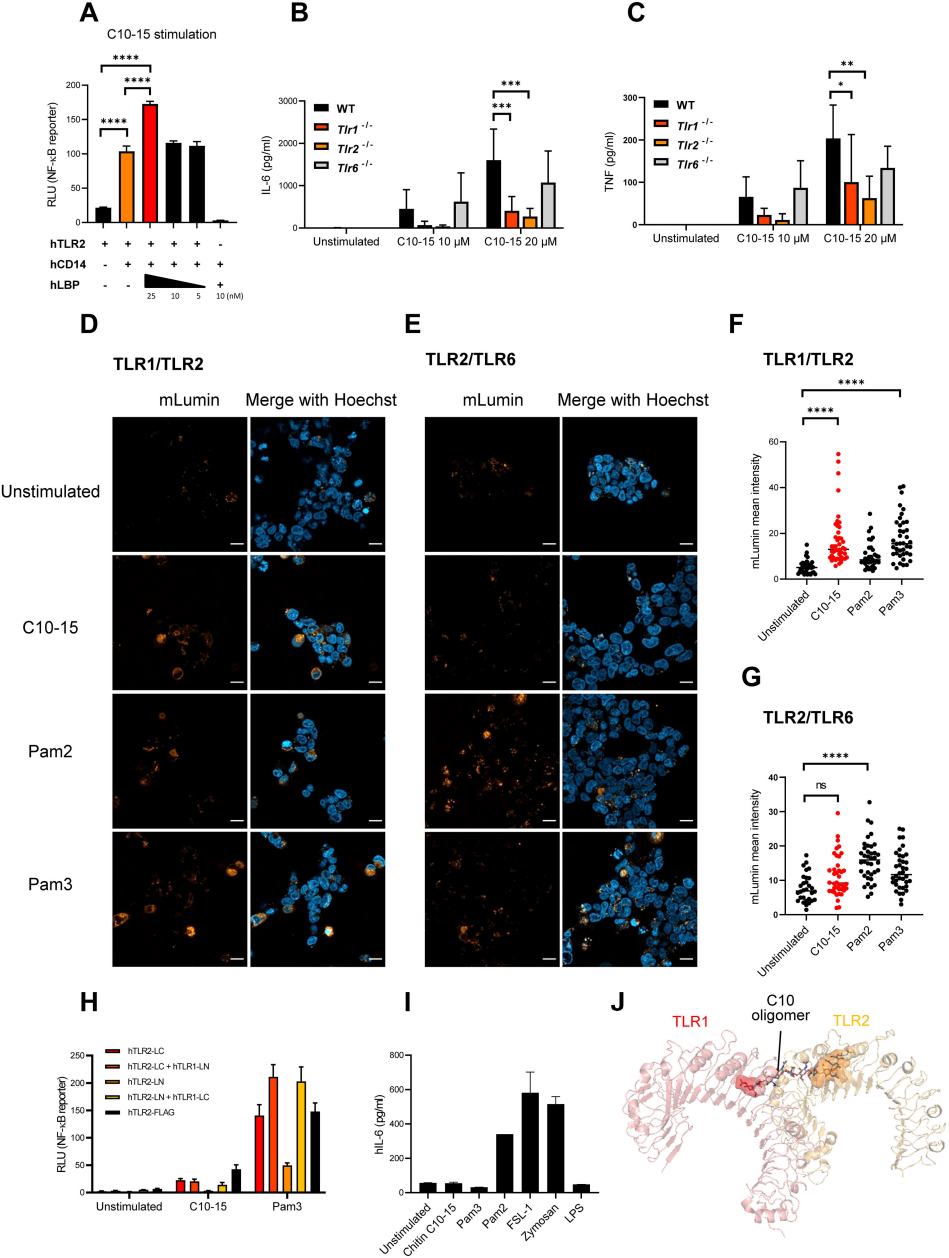
LBP, CD14, and TLR1, but not TLR6, cooperate with TLR2 to mediate oligomeric chitin recognition. **(A)** Measurement of NF-κB activity in TLR2- or TLR2/CD14-overexpressing HEK293T cells. Dose-titrated recombinant LBP protein was added together with chitin C10-15 oligomers. Cell lysates were used for dual luciferase assays conducted in technical triplicates. **(B, C)** IL-6 and TNF secretion by murine WT, *Tlr1* KO, *Tlr2* KO, and *Tlr6* KO BMDMs upon stimulation with chitin C10-15 was measured via triplicate ELISA. **(D, E)** HEK293T cells were co-transfected with split-mLumin-TLR1 and split-mLumin-TLR2 **(D)** or split-mLumin-TLR2 and split-mLumin-TLR6 **(E)**. After stimulation with Pam2, Pam3, or C10-15, cells were stained with Hoechst and inspected by confocal fluorescence microscopy. Single representative focal images from at least 20–25 cells were taken per condition. Scale bars represent 20 µm. **(F, G)** The mLumin fluorescent intensity was quantified by ImageJ. Threshold was applied to the fluorescent signal from each single cell, and the intensity was measured using the Otsu method. The mean intensity of mLumin was normalized to that of Hoechst. **(H)** Measurement of NF-κB activity in split-mLumin single- or co-transfected HEK293T cells after stimulation with C10-15 or Pam3. **(I)** IL-6 secretion in human N/TERT-1 cells upon stimulation with the indicated ligands was measured by triplicate ELISA. **(J)** Fitting of chitin decamer (stick representation) into the TLR1 (red)-TLR2 (orange) heterodimer (cartoon, hydrophobic pocket shown as surface) based on the corresponding crystal structure (PDB: 2Z7X). In **(A, D, E)** and **(I)** (n = 3 each) and **(H)** (n = 1 preliminary experiment), one representative of “n” biological replicates is shown (mean + SD for technical replicates). **(B, C)** (n = 4 each) and **(F, G)** (n = 3 each) represent combined data (mean + SD) from “n” biological replicates (in **(F, G)**, each dot represents one quantified image). *p < 0.05, **p < 0.01, ***p < 0.001, ****p < 0.0001 according to one-way **(A, F, G)** or two-way **(B, C)** ANOVA with Sidak’s **(A–C)** and Tukey’s **(F, G)** correction for multiple testing. ns, not significant; WT, wild type; KO, knockout; BMDMs, bone marrow-derived macrophages.

A prominent feature of certain innate immune signaling pathways is the involvement of soluble and membrane-bound transfer proteins such as LBP and CD14, which both promote LPS sensing via TLR4, and Pam_2_CSK_4_ (Pam2) and Pam3_3_CSK_4_ (Pam3) lipopeptide sensing via TLR2/TLR6 and TLR1/TLR2, respectively (35, 36, 50). To test whether LBP and CD14 may play a similar role in shuttling oligomeric DP10-15 chitin (C10-15, see 9), we added recombinant LBP exogenously to and/or co-expressed CD14 in TLR2–HEK293T cells before measuring NF-κB reporter activity. Evidently, CD14 overexpression strongly increased TLR2-mediated NF-κB activity, and this was further increased by the addition of LBP similar to Pam3 (**Figure 4A**; **Supplementary Figure S2**), indicating that oligomeric chitin sensing employs components of other TLR ligand sensing systems in this recognition pathway. We next tested whether oligomeric chitin TLR2 sensing also required co-receptors such as TLR1 and TLR6 (51) using anti-TLR1 and anti-TLR2 blocking antibodies in WT BMDMs (**Supplementary Figure S3A**). Evidently, the response to all TLR2 ligands (including C10-15), but not LPS, was blocked by anti-TLR2 blockade. There was a subtle effect of anti-TLR1 blocking. To verify this further, we moved to a genetic system and compared WT and *Tlr1* knockout (−/−, KO), *Tlr2* KO, or *Tlr6* KO BMDMs. Interestingly, in response to the oligomeric C10-15 chitin and Pam3, *Tlr6* KO BMDMs responded like WT BMDMs by producing IL-6 and TNF, whereas both *Tlr1* KO and *Tlr2* KO BMDMs were reduced in response to C10-15 and Pam3 (**Figures 4B, C**; **Supplementary Figures S3B, C**). This genetic analysis implicated both TLR1 and TLR2 in chitin sensing of immune cells, consistent with the earlier use of blocking antibodies directed against TLR1 or TLR6 in the HEK293T system (9). To validate TLR1 involvement further, TLR1 antibody-blocking experiments were also extended to BMDMs (**Supplementary Figure S3C**). To show that oligomeric chitin also triggered receptor dimerization as shown for TLR4 and TLR2, we conducted fluorescence complementation assays of TLR2 with the co-receptors, TLR1 or TLR6. In brief, we used expression constructs in which the TLR cytoplasmic domain is C-terminally fused to either the N- or C-terminal portion of the mLumin protein, a derivative of the far-red fluorescent protein variant of mKate (52). In this system, stable dimer formation is evidenced by the detection of mLumin fluorescence (52). In confirmation of KO and blocking results, C10-15 and Pam3 indeed induced the highest mLumin fluorescence in TLR1- and TLR2-expressing cells (**Figure 4D**), and Pam2 did so in TLR2- and TLR6-expressing cells (**Figure 4E**). Quantification of these data showed that TLR1/TLR2 co-expression induced significant mLumin fluorescence only upon stimulation with C10-15 oligomeric chitin or Pam3 (**Figure 4F**), whereas TLR2/TLR6 co-expression did so only upon stimulation with Pam2 (**Figure 4G**). Similar results were obtained for NF-κB induction in cells expressing TLR2 and co-receptors (**Figure 4H**). Additionally, we found that certain cell types, for example, N/TERT-1 keratinocyte-like cells (53), naturally responded to Pam2, whereas they failed to respond to both C10-15 and Pam3 (**Figure 4I**), indicating that the latter agonists behave similarly and that both prefer TLR1/TLR2 heterodimers. This is consistent with *in silico* analyses, which showed that a chitin decamer can be fitted into the structure of a TLR1/TLR2 heterodimer (PDB: 2Z7X (38), **Figure 4J**), whereas the absence of a hydrophobic pocket in TLR6 prevented this in a TLR2/TLR6 heterodimer structure (PDB: 3A79; 39). Collectively, our results suggest that oligomeric chitin sensing in mammals relies not only on the generation of oligomeric MAMPs by CHIT1 but also on soluble and membrane-bound co-receptors, such as CD14 and TLR1, for optimal TLR2 signal induction.

### Induction of *Chit1* expression by fungal components via Dectin-1

**Figure 5:**
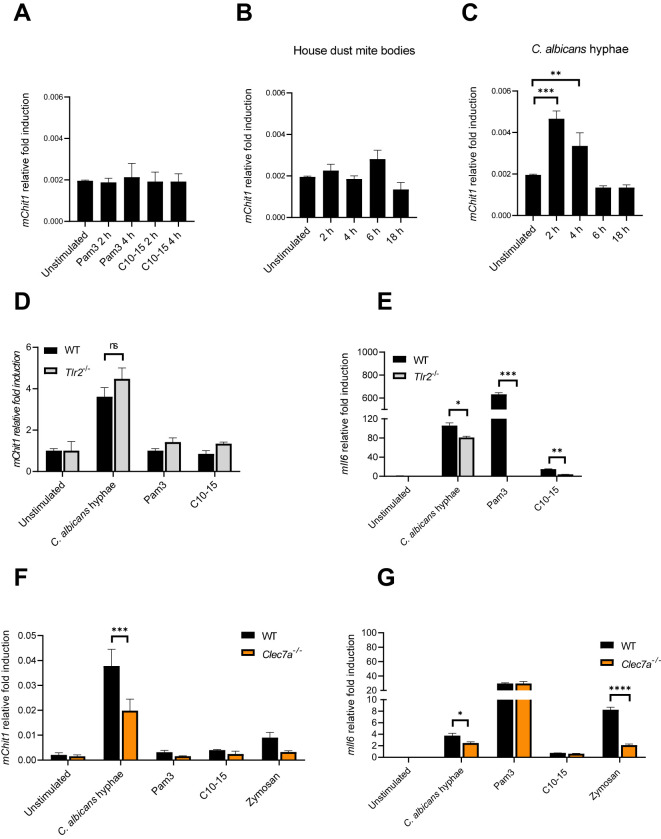
*Candida albicans* hyphae elicit *Chit1* mRNA induction via Dectin-1. **(A–C)** Fold induction of murine *Chit1* and *Il6* mRNA relative to *Tbp* mRNA in primary BMDMs upon stimulation with HDM, *C. albicans* hyphae, Pam3, and C10-15 at the indicated time points. **(D–G)** Relative fold induction of murine *Chit1* and *Il6* mRNA relative to *Tbp* mRNA in **(D, E)** WT and *Tlr2* KO primary BMDMs or **(F, G)** WT and *Clec7a* ^−/−^ iMacs upon 4 h stimulation with *C. albicans* hyphae, Pam3, C10-15, and zymosan (n = 3). In **(A–C)** (n = 4) and **(D–G)** (n = 2), one representative of “n” biological replicates is shown (mean + SD for technical replicates). *p < 0.05, **p < 0.01, ***p < 0.001, ****p < 0.0001 according to one-way ANOVA with Dunnett’s correction **(A-C)** or two-way ANOVA with Sidak’s correction **(D–G)** for multiple testing. ns, not significant; BMDMs, bone marrow-derived macrophages; HDM, house dust mite; iMacs, immortalized macrophages.

Our data thus far suggest that CHIT1 can generate diffusible, TLR2-activating oligomers from chitin-containing organisms. We therefore became interested in how CHIT1 may be regulated. In murine BMDMs, RT-qPCR analyses revealed a relatively low expression of *Chit1* compared to the housekeeping gene *Tbp* (encoding the TATA box binding protein). Exposure to oligomeric chitin (C10-15) or Pam3 did not increase *Chit1* transcription (**Figure 5A**); neither did HDM bodies (**Figure 5B**). However, *C. albicans* hyphae induced significantly more than twofold *Chit1* mRNA levels after 2-h stimulation (**Figure 5C**). The effect was transient and returned to baseline within 6 h. To test whether this induction was TLR2-dependent, WT and *Tlr2* KO BMDMs were compared. Whereas Pam3, C10-15, and *C. albicans* hyphae elicited a strong, *Tlr2*-dependent *Il6* upregulation, *C. albicans* hyphae induced *Chit1* and *Il6* in a *Tlr2*-independent manner (**Figures 5D, E**). Thus, in murine macrophages, rapid induction of *Chit1* was independent of TLR2 and oligomeric chitin. These results are consistent with the fact that, in fungal cell walls, chitin is initially buried under β-glucan and mannoproteins (1, 54). At first encounter with a fungal pathogen, there would thus be little accessible chitin, and an induction of chitinase activity via another MAMP such as β-glucan would, hence, be more plausible. Therefore, we speculated that *Chit1* mRNA induction by *C. albicans*—which is consistent with earlier analyses using macromolecular chitin in primary macrophages (9)—could be mediated by β-glucan sensing through Dectin-1 (encoded by *Clec7A*; 55). We compared the expression of *Chit1* and *Il6* using RT-qPCR analysis in mRNA extracted from WT and *Clec7a* KO immortalized macrophages (9, 56) stimulated with *C. albicans*, Pam3, C10-15, and zymosan, a yeast cell wall extract sensed through TLR2 and Dectin-1 (9). Interestingly, soluble TLR2 agonists (Pam3 and C10-15) did not show differences between cell types for *Chit1* and *Il6* transcription (**Figures 5F, G**). However, *C. albicans* and zymosan induced drastically less *Chit1* and *Il6* by *Clec7a* KO macrophages (**Figures 5F, G**). These data indicate that Dectin-1 may be responsible for inducing the expression of *Chit1* in response to *C. albicans*. This would be consistent with β-glucan being a major and more readily exposed fungal MAMP (55) that may prime CHIT1-mediated oligomeric chitin MAMP generation.

### CHIT1 is degraded by the fungal proteases Sap2 and Sap6

**Figure 6:**
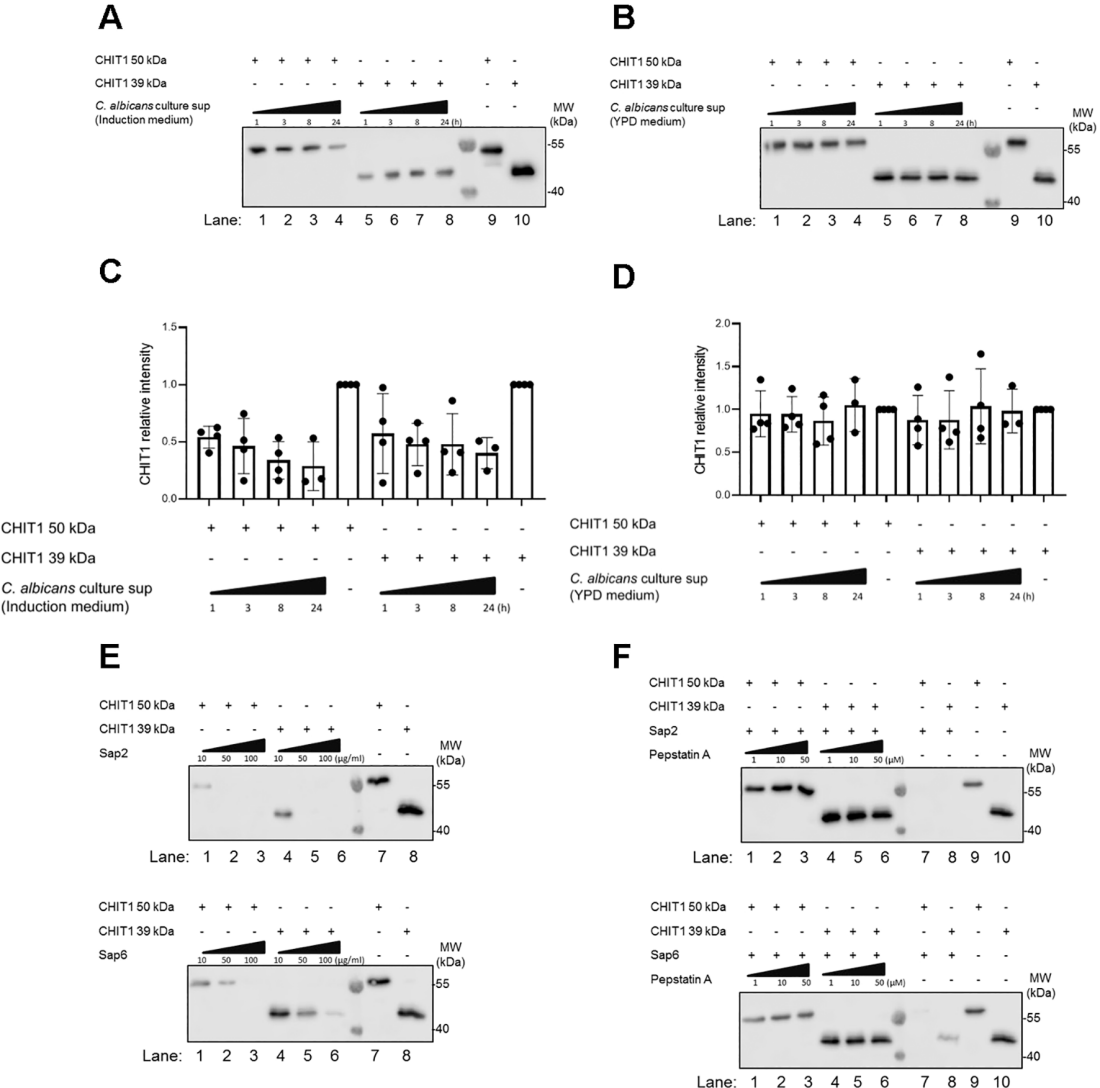
CHIT1 is degraded by secreted *Candida albicans* proteases. **(A, B)** *C*. *albicans* was cultured for 48 h in protease induction medium **(A)** or YPD medium **(B)**. *C*. *albicans* culture supernatants were then collected and incubated with His-tagged variants of CHIT1 50 kDa or 39 kDa for the indicated time points. Recombinant CHIT1 isoforms (His-tagged) were detected by immunoblot with an anti-His antibody. CHIT1 isoforms without any treatment were used as positive controls. **(C, D)** CHIT1 anti-His relative intensities were quantified and normalized to untreated conditions. **(E)** Recombinant CHIT1 isoforms (His-tagged) were treated with or without the indicated doses of recombinant Sap2 (upper panel) or Sap6 (lower panel) for 8 h and detected as in panel **(A, F)** As in **(E)** but with 50 µg/mL Sap2 or Sap6 with or without the indicated doses of pepstatin A aspartic protease inhibitor. In **(A–C)** (n = 4 each) and F (n = 2), one representative of “n” biological replicates is shown (mean + SD for technical replicates). **(C)** and **(D)** (n = 4 each) represent combined data (mean + SD) from “n” biological replicates (each dot represents one biological replicate). YPD, yeast extract–peptone–dextrose.

Based on these results, increasing CHIT1 activity and the subsequent emergence of diffusible TLR2 ligands during infection over time would mean an increasing contribution of TLR2-dependent immunity to antifungal responses, which have been demonstrated in infection models (18, 33). This would make CHIT1 a potential target for fungal counterstrategies. Indeed, in plants, chitinases have been shown to represent proteolytic targets of fungal proteases (5). Since *C. albicans* can express multiple secreted aspartyl proteinases (Saps) (57), we tested whether *C. albicans* culture supernatants contained proteolytic activity to which CHIT1 may be sensitive. We incubated (His-tagged) recombinant CHIT1 preparations with fungal supernatants from *C. albicans* grown under conditions known to induce Sap secretion (in “induction media”, 58). This led to a decrease of anti-His signal, i.e., CHIT1 protein levels (**Figure 6A**), when compared to samples without *C. albicans* supernatant (lanes 9 and 10 in **Figure 6A**). Degradation was not observed when *C. albicans* supernatant was harvested from fungal cultures that had been grown in standard yeast extract–peptone–dextrose (YPD) media (**Figure 6B**), where Saps are only expressed at low levels (58). This was confirmed by immunoblot quantification analysis from several experiments (**Figures 6C, D**). These results indicate that under these given conditions, *C. albicans* cells may secrete Saps to target CHIT1. To test this hypothesis further, CHIT1 was also exposed to purified recombinant *C. albicans* Sap2 and Sap6, representing Saps expressed by yeast and hyphae, respectively. Both 50-kDa and 39-kDa forms of CHIT1 were dose-dependent on the proteolytic activity of Sap2 and Sap6 (**Figure 6E**), as the effect was blocked by the aspartic protease inhibitor pepstatin A (**Figure 6F**). Thus, Chit1 is sensitive to the fungal virulence factors and proteases Sap2 and Sap6 and may therefore be targeted by the pathogenic fungus, *C. albicans*.

## Discussion

**Figure 7:**
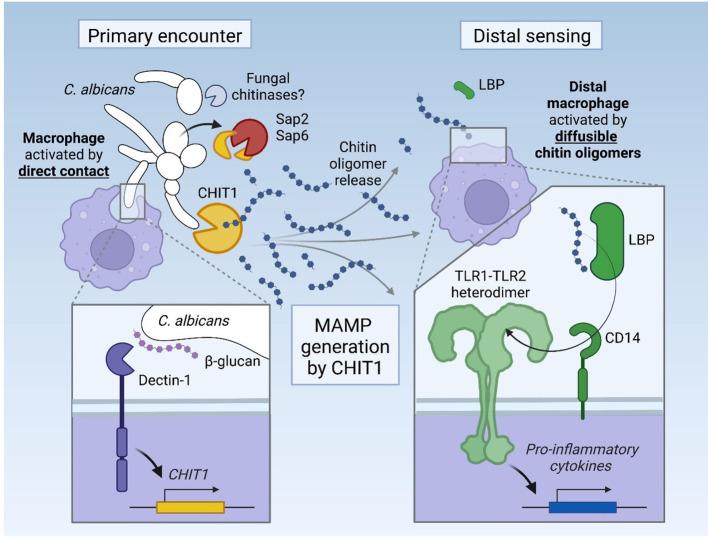
Graphical abstract. Upon its induction via Dectin-1, CHIT1 releases diffusible oligomeric chitin from chitin-bearing organisms, which can trigger remote macrophages via an LBP–CD14–TLR1/TLR2 signaling cascade. The activity of CHIT1 may be counteracted by fungal proteases Sap2 and Sap6, which can degrade CHIT1.

Here, we uncover several new facets in the immune sensing of diffusible chitin oligomers. In addition to direct, contact-mediated sensing of chitin-containing structures by TLR2 (9), our data highlight CHIT1 as a host factor able to generate diffusible TLR2-cognate MAMPs, and LBP and CD14 as important soluble components of this sensing pathway (**Figure 7**). Furthermore, our data establish TLR1 as a co-receptor for cell surface sensing of the fungal MAMP chitin. The similarities of this system to TLR4 sensing (reviewed in 35) are unexpected and more striking than previously envisaged: first, our data indicate chitin sensing, like LPS sensing, similarly requires circulating MAMP “extractors” and “shuttles”, LBP (35, 36) and CD14 (50, 59, 60), probably owing to the same hydrophobic nature of the two MAMPs, LPS and chitin. LPS can be extracted from LPS-bearing microbes as a monomer (36), whereas chitin first requires degradation by CHIT1 to give rise to immunostimulatory MAMPs. Unexpectedly, the 39-kDa protein emerged as more efficient at generating stimulatory TLR2 ligands despite binding less to chitin, as has been reported (27). We speculate that the 39-kDa protein would thus be expected to degrade macroscopic chitin with a higher chance that the initially released oligomers in a DP range conducive for TLR2 activity (DP > 6) are not cut down below DP6 at which point TLR2 activity is lost (see 9). The 50-kDa form, however, would be more likely to remain associated with an already cleaved oligomer for further processing or to bind via its CBD and cleave an already generated small oligomer another time. Although this would be useful for physically degrading pathogenic microbes faster, it entails a lower generation of suitable TLR2 ligands. The reported proteolytic cleavage of the 50-kDa form into a 39-kDa form (27) may thus be a mechanism to ensure that sufficient TLR2 ligands are also generated, especially in tissues, where the 39-kDa dominates.

MAMP generation in the host had previously only been shown for *Drosophila* and plants, suggesting that the mammalian chitin–CHIT1–LBP–CD14–TLR1/TLR2 cascade resembles the pattern recognition mechanism in *Drosophila* even more closely than the LPS–LBP–CD14–MD2–TLR4 pathway in mammals. In *Drosophila*, certain soluble peptidoglycan recognition proteins (PGRPs), the main sensors for bacteria in flies, are hydrolases degrading peptidoglycan. Depending on the receptor involved, peptidoglycan degradation was shown to downregulate or induce host responses (61, 62). GNBP1 can also hydrolyze peptidoglycan for recognition by soluble PGRP-SA (12), triggering an extracellular proteolytic cascade ultimately leading to the cleavage of Spätzle, the *Drosophila* Toll receptor ligand (63). GNBP1 has also been suggested to sense β-1,3-glucans (64) and would thus fulfill the role of a protein binding polymeric MAMPs in flies, whereas our work identifies CHIT1 as a human polymeric MAMP binder and simultaneous “MAMP generase” for a specific PRR. Strikingly, the similarity also extends to the level of protein domains, as both CHIT1 and GNBPs possess carbohydrate-binding and glycoside hydrolase domains. Finally, similarities extend even to the plant kingdom, in which chitin-degrading enzymes generate oligomeric chitin ligands (5, 65). Despite small species-specific differences, our work thus defines a unified concept in fungal recognition, involving circulating MAMP generases, shuttles, and linked PRRs in plant and animal kingdoms.

The importance of this process for fungal recognition in humans is underscored by evidence that *C. albicans* can target this pathway at the level of MAMP generation through protease activity, reminiscent of counterstrategies used by fungi to subvert immune responses in plants (5). For example, *Paracoccidioides brasiliensis* utilizes its own chitinases to generate immunomodulatory chito-oligomers (66), whereas the plant pathogens *Fusarium oxysporum* and *Ustilago maydis* secrete metalloproteases and/or serine proteases that cleave and thereby inactivate chitinases of tomato and maize, respectively (5). During the infection of mammalian hosts, fungal cell wall remodeling affecting chitin exposure may occur as a consequence of changes in carbon availability, pH, exposure to antifungal drugs, and the host immune response (67–72). Targeting circulating CHIT1 via soluble effectors may be an additional way for pathogenic fungi to avoid subsequent chitin recognition through TLR2. Although Saps degrade multiple host proteins and are thus not exclusive for CHIT1 (73), our findings may nevertheless explain why no fungal counter-strategies against TLR2 signaling have been described so far since targeting the upstream MAMP generase CHIT1 may be more favorable. In agreement with our data, patients with *CHIT1* deficiency-associated polymorphisms have higher colonization levels by *C. albicans* (32) and an increased risk of fungal infection by *Madurella mycetomatis*, the causative agent of mycetoma (74). Our data suggest that impaired TLR2 MAMP generation may contribute to a clinical phenotype of *CHIT1* deficiency in these patients.

Despite the possibility of CHIT1 being targeted by fungi, we suggest that employment of a humoral MAMP generase has advantages for the host over direct recognition of chitin by cell surface receptors. Not only would CHIT1 generate numerous oligomers from a single microbe, but the generation of diffusible MAMPs would also overcome the need for innate immune cells to physically engage chitin-bearing microorganisms. For example, phagocytosis and Dectin-1/TLR2-mediated TNF production are critical host responses upon direct engagement of *C. albicans* and other pathogenic fungi (75). However, the requirement of direct contact would limit responsiveness to locally resident immune or tissue cells and would preclude sensing of MAMPs by distal cells and tissues. Beyond local, contact-based immunostimulation, the release of diffusible, oligomeric chitin by CHIT1 enables TLR2 sensing of fungal MAMPs at a distance, providing a means for the attraction or recruitment of further immune cells and upregulation of antimicrobial defenses. This may be of particular relevance in epithelia such as the lung, where CHIT1 is expressed (20) and where a surface area of 180–200 m^2^ (76) requires constant immune surveillance. Here, a sensing system based on diffusible MAMPs is highly advantageous because it requires a far lower number of sentinel immune cells than if immune sensing only relied on direct contact. Interestingly, our preliminary data suggest that CHIT1 expression may be induced upon immune sensing of fungi via Dectin-1. Such a sophisticated regulatory mechanism would allow to amplify host innate immune responses by broadening the panel of MAMPs for both local and distal sensing.

Aside from the detection of pathogenic fungi, our data indicate that the link between CHIT1 and the TLR2 sensing system may also be relevant in the context of HDM allergies, where CHIT1-generated chitin oligomers could serve as TLR2-acting adjuvants for HDM antigens. An intriguing link to be explored relates to the Der p 18 allergen from *D. pteronyssinus*: this protein shares a GH18 hydrolase fold, chitin-binding capability, and several conserved GH18 active site residues with CHIT1 (44, 77) and thus may add to oligomer generation. Further analyses of the enzymatic activity and physiological relevance of this and other microbiome-encoded GH18 family members are hence warranted. Our results support the notion that CHIT1 enzymatic activity may be an advantageous target for intervention in HDM–chitin–TLR2-mediated pathologies such as allergic asthma or the skin condition rosacea (78–80). Interestingly, preclinical studies using CHIT1 inhibitors are underway for the prevention of lung fibrosis (81, 82) and may also be efficacious in preventing the generation of inflammatory TLR2 ligands *in situ*. However, given the abovementioned disparate results obtained in experimental murine *in vivo* models of *Chit1* deficiency and the differences in expression between the mouse versus human proteins (83), an extrapolation from our *in vitro* data and these mouse models to an application in patients is difficult. We also acknowledge the exclusive use of *in vitro* systems as a significant limitation of this present work. However, it would have been unethical to repeat animal experiments after the effect of the key factors investigated here in the context of fungal infection has already been established *in vivo*: for example, in an *in vivo* model of systemic candidiasis, Chit1 contributed to host resistance in mice (18), consistent with genetic chitinase deficiency of *CHIT1* to associate with *C. albicans* colonization in patients with cystic fibrosis (32). Additionally, CD14, TLR1, and TLR2 have been investigated in fungal infection models before (84–87). The role of CHIT1 or TLR2 was also investigated in HDM-mediated asthma in *in vivo* animal models or human association studies (26, 78, 88, 89), albeit, like most of the aforementioned *in vivo* studies, lacking mechanistic insights for which our study here now provides rational connecting framework. We acknowledge that we also did not investigate direct antimicrobial effects of CHIT1 on fungal pathogens because CHIT1 is known to inhibit growth of *C. albicans* hyphae and that ectopic expression of CHIT1 in hamster ovary cells restricted the growth of *C. albicans*, *Aspergillus niger*, and *Cryptococcus neoformans* (30). A contribution to macrophage activation via chitin or chitosan degradation had been noted (31) but was never connected to the TLR2 sensing system or compared to fungal sensing in other species.

In conclusion, our data show that CHIT1 can serve as a human MAMP generase for the TLR2 chitin sensing cascade and thereby reveal an astonishingly high level of similarity in how fungal pathogens are sensed in species as different as humans and plants.

## Materials and methods

### Reagents and the quality control of chitin

All chemicals used in the lab were from Sigma-Aldrich unless otherwise stated. The source and origin of all the ligands, recombinant proteins, and inhibitors are listed in **Supplementary Table S1**. The preparation of C10-15 chitin oligomers has been described before (9). In brief, C10-15 chitin oligomers were generated from C10-15 chitosan (2,000–3,000 MW, equivalent to 10–15 subunits, Carbosynth), which was derived from crab shells, chemically hydrolyzed and High performance liquid chromatography (HPLC)-fractionated to >95% purity as confirmed by HPLC and mass spectrometry analysis; see Fuchs et al. (9) for further details. Using sodium bicarbonate and acetic anhydride acetylation (90), chitosan was acetylated to chitin. The resulting degree of acetylation was assessed upon trifluoroacetic acid hydrolysis (for C10-15, 2 h at 100°C) by Electrospray ionization (ESI) and Matrix-assisted laser desorption/ionization (MALDI) mass spectrometry; see Fuchs et al. (9) for further details. Only batches with up to 90% of acetylation were used here. Prior to using sterile stimulation, acetylated chitin oligomers were suspended in endotoxin-free water and tested for endotoxin level using the limulus amebocyte lysate (LAL) assay (Lonza, CH). Levels below 0.25 EU/mL (<25 pg/mL LPS) in final dilutions were considered acceptable. For levels >0.25 EU/mL, the chitin preparation was incubated for 3 h with 10 µg/mL of the LPS binding agent polymyxin B (Thermo Fisher), washed by centrifugation, and re-assessed.

### Plasmid constructs

The plasmids used in this study are listed in **Supplementary Table S2**. Gateway-compatible split-mLumin backbone plasmids for bimolecular complementary assay were described in Weiler et al. (91). Full-length *TLR1* and *TLR2* Open reading frame (ORF)-containing Gateway Entry plasmids were obtained from the PlasmID Repository at Harvard Medical School. With respect to *TLR6*, a full-length ORF flanked with Gateway *attB* sites was synthesized by GENEWIZ and transferred by BP Clonase reaction into an *attL* site-containing Gateway Entry plasmid. To transfer the hTLR1, hTLR2, and hTLR6 ORFs into the split-mLumin N-terminal and C-terminal Gateway Destination vectors, LR reactions using LR Clonase II Enzyme mix kit (Thermo Fisher) were used. The destination vectors were validated by restriction enzyme *Bsg*I (New England Biolabs) (200–300 ng plasmid and 5 units of enzyme in 1X Tango Buffer, 1 h at 37°C water bath) and Sanger sequencing (GATC Biotech). After sequencing, correct protein expression was verified by Western blotting (see details below). Plasmids encoding the 50- and 39-kDa forms of *CHIT1* as C-terminally His-tagged constructs have been described before (43) and used exactly as described. To simultaneously introduce the two point mutations D138A and E140L in the catalytic site of the chitinase, the QuikChange II XL site-directed mutagenesis kit (Agilent) was used together with mutagenesis primers (**Supplementary Table S3**) designed by following the QuikChange manufacturer’s instruction and verified *in silico* using the Geneious R6 software (6.1.8 version). The presence of the desired mutation and the absence of unwanted mutations were confirmed by Sanger sequencing (GATC Biotech). Secretion of the proteins was confirmed by immunoblot, and the catalytic inactivity of the mutant CHIT1 proteins was checked by measuring chitinase activity.

### *C. albicans* maintenance and growth conditions

*C. albicans* strain SC5314 was used in this study, in which the stock was a kind gift from Dr. Anurag Singh (Universitätsklinikum Tübingen), and stored frozen at −80°C in RPMI medium only (without Fetal calf serum (FCS)) containing 20% glycerol. When needed, cells were taken up from the frozen stock and grown at 30°C in YPD agar medium [1% (w/v) BactoYeast extract, 2% (w/v) BactoPeptone, 2% (w/v) dextrose, and 2% (w/v) agar] on 10-cm dishes. After overnight incubation, cells were stored at 4°C for up to 1 month. Before any experiment or treatment, cells were freshly prepared by sub-culturing from a 10-cm dish to a glass slant tube at 30°C. Cells were harvested by picking up a smear and re-suspending it in RPMI-1640 medium. To expose the chitin content on the surface of *C. albicans*, yeast cells were incubated with 0.032 µg/mL caspofungin (Sigma-Aldrich) for 6 h at 30°C in RPMI-1640 medium. After incubation, yeast cells were washed twice with Dulbecco's Phosphate Buffered Saline (dPBS) and tested once for viability. For hyphal induction, 1 × 10^6^ live yeast cells were re-suspended in YPD medium with 20% FCS and incubated at 37°C for at least 3.5 h until 90%–95% filamentation was observed. The hyphae were collected via a cell scraper and then washed twice with dPBS. For heat killing, both *C. albicans* yeast and hyphae were prepared by incubation at 65°C in a water bath for 1 h, with killing confirmed by plating in a YPD agar slant tube. For Saps, *C. albicans* was cultured in an induction medium (2 mM MgSO_4_, 7.3 mM KH_2_PO_4_, 1% glucose, 0.5% bovine serum albumin, and 1% 100× HEPES, pH 4.0) for 48 h at 30°C as described previously (58). The culture supernatants were used to incubate with recombinant CHIT1 and to test the degradation effect by immunoblot.

### Recombinant human CHIT1 and *C. albicans* Sap2 and Sap6

Recombinant 50-kDa and 39-kDa CHIT1 proteins were expressed as C-terminally 8xHis-tagged constructs in HEK293-6E cells and purified from the cell culture supernatant by His-Trap (GE Healthcare) affinity as described in Stockinger et al. (43). *C. albicans* Sap2 and Sap6 were expressed in *Pichia pastoris* and purified via ion exchange chromatography and size exclusion as described in Schild et al. (92).

### House dust mite and chitin flake preparation

House dust mite *D. pteronyssinus* whole cultures and mite bodies were obtained from CITEQ Biologics. The powder of whole cultures and mite bodies was weighed and re-suspended in dPBS. Polymyxin B (Thermo Fisher) was then applied to a final working concentration of 100 units/mL to remove endotoxin. After incubation for at least 1 h at Room temperature (RT), whole cultures and mite bodies were re-suspended in dPBS to 10 mg/mL (stock concentration) and stored at −20°C. Whole cultures and mite bodies at a working concentration of 100 µg/mL were used. Chitin flakes from shrimp shells were bought from Sigma-Aldrich (Catalog # C9213-500G). The flakes were first sieved using 2-mm or 1-mm pore size steel sieves (Amazon) to sort small pieces of flakes (**Supplementary Figure S1A**). The obtained flakes were picked up by clean forceps into sterile 1.5-mL Eppendorf tubes and treated with polymyxin B (10 µg/mL) for 3 h at RT. The flakes were washed three times with dPBS, finally re-suspended into 1 mL dPBS, and stored at 4°C.

### CHIT1 digestion of chitin particles

Chitin flakes, *C. albicans* preparations, house dust mite whole cultures, and mite bodies were incubated with human recombinant CHIT1 for digestion. Both 50-kDa and 39-kDa recombinant CHIT1 (stock solution 1 µM) were used at a working concentration of 4 nM (1:250 dilution). For expression of WT or mutant CHIT1-encoding plasmids in HEK293T cells, 250 ng of *CHIT1* plasmids was transfected into HEK293T cells supplemented with 250 µL Dulbecco's Modified Eagle Medium (DMEM) complete medium. After 48-h transfection, the collected cultured medium was used for chitinase digestion. The secretion of both CHIT1 isoforms was confirmed by immunoblot. To digest chitin flakes, *C. albicans* preparations, house dust mite whole cultures, and mite bodies were incubated with recombinant or secreted CHIT1-containing media in 1.5 mL Eppendorf tubes on a rotation wheel for 18 h at RT. After CHIT1 digestion, the culture supernatants were used for stimulation or applied to transwells.

### HEK-Dual™ hTLR2 (NF-κB/IL8) reporter cells

HEK-Dual™ hTLR2 cells were bought from InvivoGen. The cells were derived from human embryonic kidney 293 (HEK293) and stably transfected with the human *TLR2* gene, an NF-κB/AP-1 inducible secreted embryonic alkaline phosphatase (SEAP) reporter construct, and a Lucia luciferase (a secreted luciferase), inserted under the control of the endogenous human *IL8* promoter. Cells were kept under the antibiotic selection of Hygromycin B and Zeocin.

### Immortalized macrophage culture and differentiation

Macrophage progenitors derived from the bone marrow of C57BL/6 wild-type and *Clec7a* ^−/−^ mice were immortalized as described (56) and a kind gift of Philip Taylor, Cardiff University. Progenitor cells were kept and cultured in the RPMI-1640 complete medium supplemented with 1 µM (β-estradiol) and 10% murine granulocyte-macrophage colony-stimulating factor (mGM-CSF). Progenitor cells were split 1:10 depending on demand and maintained for less than 10 passages. Before differentiation, progenitor cells were washed and centrifuged twice with dPBS to remove β-estradiol. A density of 5 × 10^5^ progenitor cells was then seeded in a 12-well plate with 1.5 mL RPMI-1640 complete medium supplemented with 10% mGM-CSF without β-estradiol. An additional 1 mL of fresh culture medium was added on day 2. On day 5 of differentiation, adherent cells as differentiated macrophages were harvested and plated in 12-well plates (2 × 10^6^ cells/well) in RPMI-1640 complete medium without mGM-CSF. After 1 day, the cells were ready for stimulation.

### N/TERT-1 cell culture

N/TERT-1 cells were a gift from Prof. James Rheinwald (53) and acquired from local collaborator Birgit Schittek, Dermatology Department, University Hospital Tübingen. Cells were passaged and maintained in CnT-07 medium (Epithelial Proliferation Medium, from CELLnTEC, Catalog # CnT-07) for less than 10 passages. Before stimulation, N/TERT-1 cells were seeded at a 96-well plate (2 × 10^4^ cells/well) in CnT-07 medium. After 2 days of resting, cells were ready for stimulation. After 24 h of stimulation, supernatants were subjected to ELISA according to the manufacturer’s instructions (BioLegend).

### Mice and primary bone marrow-derived macrophages

C57BL/6 wild-type and *Tlr2* KO mice on a C57BL/6 background (originally a gift from H. Wagner, Ludwigs-Maximilian University, Munich) were maintained in the animal facility of the Department of Immunology, University of Tübingen, used between 8 and 20 weeks of age, and sacrificed using CO_2_. Animal breeding, handling, and sacrificing between 8 and 20 weeks of age were performed according to the local institutional guidelines and institutionally approved protocols. WT, *Tlr1* KO, *Tlr2* KO, and *Tlr6* KO mice on a C57BL/6J background have been described before (93) and maintained at the animal facility of Lausanne University Hospital, Lausanne, Switzerland. Animal experiments performed in Lausanne were approved by the Service des Affaires Vétérinaires, Direction Générale de l’Agriculture, de la Viticulture et des Affaires Vétérinaires, état de Vaud (Epalinges, Switzerland) under authorizations 876.9 and 3587 and performed according to Swiss and ARRIVE guidelines. Bone marrow cells were isolated from femurs and tibias, which were cut and flushed out using a 24-gauge syringe, and re-suspended in RPMI-1640 medium supplemented with 10% FCS. A density of 1.2 × 10^7^ cells was seeded in 10-cm Petri dishes in RPMI-1640 complete medium containing 10% culture supernatant from mGM-CSF-producing cells (94). An additional 5 mL of fresh culture medium was added on day 3. On day 7 after differentiation, adherent cells were harvested and plated in 96-well plates (1 × 10^5^ cells/well) or 12-well plates (2 × 10^6^ cells/well) in RPMI-1640 complete medium without mGM-CSF. After 1 day of resting, cells were ready for stimulation.

### Transwell setting

Transwell inserts for 24-well plates (8-µm pore size, Nunc) were used, and 250 µL of media containing chitinase-digested chitin flakes, *C. albicans*, or house dust mite bodies was filled into the upper reservoir, which was placed onto the cell culture plate containing 250 µL media and TLR2-transfected HEK cells or BMDMs at its bottom. After 18-h incubation, almost two-thirds of the culture medium from the layer of transwell eventually passed through to the cell culture plate. The transwell was then removed and checked under the microscope. Large particles like chitin flakes and house dust mites were always still trapped in the upper part of the transwell insert. For a schematic impression, see **Supplementary Figure S1B**. For *C. albicans*, minor amounts of yeast cells or hyphae were sometimes found in the lower compartment after incubation. Cells or culture supernatants were processed as described for the respective assays.

### BMDM analysis with TLR1 and TLR2 blocking antibodies

Experiments using blocking antibodies were conducted as described previously (9). In brief, 5 × 10^4^ BMDM cells per well were seeded in RPMI media supplemented with 10% Fetal bovine serum (FBS) in 96-well plates. After 1 h of seeding, TLR1 or TLR2 blocking antibodies (InvivoGen) were added to a final concentration of 4 µg/mL, and cells were incubated for 1 h at 37°C, 5% CO_2_—Pam2 (40 nM), Pam3 (40 nM), and LPS EB (40 nM; all from InvivoGen) or C10-15 (5 µM)—and incubated for 24 h. Isotypes were not included, as these commercially available reagents have been validated before, and LPS was included as a specificity control. Supernatants were then collected and measured for ELISA.

### Dual NF-κB luciferase assay in HEK293T cells

An inoculum of 5 × 10^4^ HEK293T cells was seeded in a 24-well plate with 500 µL DMEM complete medium and incubated overnight. The next day, cells were transfected with the following amounts of plasmid DNA per well: 100 ng NF-κB firefly reporter luciferase and 10 ng of *Renilla* luciferase under a constitutive promoter. To measure the TLR2 response, cells were transfected with 100 ng of human TLR2 or with 100 ng backbone as an empty vector control as described (9). Transfection was performed using 1 mL of X-tremeGENE™ HP DNA Transfection Reagent (Sigma-Aldrich) mixed into a total of 50 mL Opti-MEM together with the above-indicated plasmids. After 15 min of incubation at RT, 50 mL Opti-MEM-X-treme/plasmid mixture was added dropwise onto the cells. After 48 h of incubation, the medium was replaced by fresh DMEM complete medium with or without TLR agonists. Cells were stimulated for 18 h, and cell lysates were harvested. The concentrations of all stimuli and ligands are listed in **Supplementary Table S1**. To analyze luciferase activity, the culture supernatants were removed, and the cells were washed with 350 mL dPBS. Cells were lysed in 60 mL passive lysis buffer (Promega). After shaking for 5 min, cells were frozen at −80°C for at least 15 min. Thawed lysates were cleared by centrifugation, and 10 mL of the cleared lysate was used to measure the luciferase activity on a FluoStar plate reader (BMG Labtech). In the plate reader, the substrates for firefly and *Renilla* luciferase were automatically injected. The analysis settings were used as recommended in the luciferase reporter system by Promega. The results were calculated as the ratio between firefly luminescence and *Renilla* luminescence (**Figures 1A–F**, **4A, H**; **Supplementary Figure S2**).

### HEK-Dual™ hTLR2 (NF/IL8) reporter cell assay

Initially, 5 × 10^4^ HEK-Dual™ hTLR2 cells (InvivoGen) cells were seeded in a 96-well plate. After overnight incubation, the culture medium was exchanged with fresh DMEM complete medium with or without the stimuli. After 18 h, the cell culture supernatant was analyzed for NF-κB/AP-1-induced SEAP production using QUANTI-Blue reagents and IL-8-dependent expression of Lucia luciferase using QUANTI-Luc reagents (both InvivoGen). For QUANTI-Blue measurement, 20 µL of cell culture supernatant was added to 180 µL QUANTI-Blue solution in a 96-well flat plate. The plate was incubated for 30 min at 37°C. The SEAP levels were determined by a SpectraMax^®^ plate reader at 650 nm. For QUANTI-Luc measurement, 10 µL of cell culture supernatant was pipetted in a 96-well white plate. Lucia luciferase activity was measured using a FluoStar plate reader (BMG Labtech), which automatically added 50 µL of QUANTI-Luc solution. Data in **Figures 1G, H** were generated using this reporter assay.

### Immunoblotting

Protein expression in HEK cells transfected with chitotriosidase- or TLR-encoding plasmids was verified by immunoblotting. In order to detect secreted chitotriosidase, after 24-h transfection and a further 24-h incubation in fresh Opti-MEM without any FCS, both whole cell lysates (WCL) and culture supernatants were harvested. For obtaining WCL, cells were washed once with dPBS and then lysed with 60 mL RIPA buffer (20 mM Tris-HCl, pH 7.4, 150 mM NaCl, 1 mM Ethylenediaminetetraacetic acid (EDTA), 10% glycerol, 0.1% Sodium dodecyl-sulfate (SDS), 1% Triton X-100, and 0.5% deoxycholate) supplemented with EDTA-free protease inhibitor (Roche) and 0.1 mM Phenylmethylsulfonyl fluoride (PMSF). After maximum speed centrifugation, WCL were mixed with reducing reagent and sample loading buffer (Novex, Thermo Fisher) and then boiled for 5 min at 95°C to denature the proteins. For obtaining the supernatants, 60 mL of supernatants was treated as described for WCL. Samples (10 mL of WCL and 20 mL of supernatant) were subjected to electrophoresis on 10% Tris-glycine gels with SDS buffer (25 mM Tris-base, 250 mM glycine, and 0.1% SDS) and transferred to a 0.45-mm nitrocellulose membrane (GE Healthcare). The membrane was blocked with 5% bovine serum albumin (BSA) (w/v) in Tris-buffered saline solution with 0.1% (v/v) Tween-20 (TBS-T) for 1 h at RT and then left in 5 mL buffer containing primary antibody (see **Supplementary Table S4**) in 5% BSA in TBS-T overnight at 4°C with rotation. The next day, the membranes were washed three times with TBS-T for at least 5 min and then incubated with 10 mL TBS-T buffer containing secondary antibody in 5% non-fat milk for 1 h at RT with rotation. After incubation with secondary antibodies, membranes were washed three times with TBS-T for at least 5 min per wash. ECL reagent (Peqlab) was used to detect chemiluminescence, and the development was performed using a Licor camera Odyssey Imaging system in the chemiluminescence channel. Pictures were analyzed and edited in the Image Studio Lite software. The relative intensities of CHIT1 and His expression were also quantified by Image Studio. For the verification of TLR1, TLR2, and TLR6 constructs, TLR protein expression was verified using anti-TLR1, anti-TLR2, and anti-TLR6 as primary antibodies (**Supplementary Table S4**). For whole protein staining, after transfer, nitrocellulose membranes were first rinsed with ddH_2_O and then incubated with 5 mL Revert 700 Total Protein Stain Solution (Licor) for 5 min. After washing twice with 5 mL Wash Solution, the membranes were subjected to the Licor Odyssey Imaging system in the 700-nm channel. After imaging, the membranes were destained with 5 mL Revert Destaining Solution for 5–10 min until the stain was no longer visible to the eyes. The destained membranes were washed twice with ddH_2_O, and the 5% BSA blocking was applied immediately.

### Measurement of chitinase activity

Chitinase activity in the culture supernatant of *CHIT1* plasmid-transfected HEK293T cells was measured using a fluorescence assay kit (Sigma-Aldrich, Catalog # CS1030). A 10-mL supernatant sample was mixed with 90 mL substrate solution containing 0.5 mg/mL of 4-methylumbelliferyl *N*,*N*′-diacetyl-β-d-chitobioside in an assay buffer provided as part of the kit and in a 96-well plate (black/clear bottom plate; Thermo Fisher). The reaction was incubated at 37°C for 30 min and then stopped by adding 100 mL stop solution. The fluorescence was measured at excitation of 360 nm and emission of 450 nm on a FluoStar plate reader (BMG Labtech).

### ELISA

Appropriate ELISA kits were used to quantify human or murine IL-6 and/or TNF concentrations according to the manufacturer’s recommendations (BioLegend). The 450-nm absorbance was measured using a SpectraMax^®^ plate reader.

### Split-mLumin-based bimolecular fluorescence complementation assay

Suspensions of 2 × 10^4^ HEK293T cells were seeded in 8-well 1.5H glass chamber slides (Ibidi). After 1 day of resting, cells were co-transfected with split-mLumin plasmids in the following two listed combinations: 1) hTLR2-LC151 (200 ng; mLumin C-terminal fragment) + hTLR2-LN151 (200 ng; mLumin N-terminal fragment) and 2) hTLR2-LN151 (100 ng; mLumin N-terminal fragment) + hTLR6-LC151 (300 ng; mLumin C-terminal fragment). After 48-h transfection, cells were stimulated with or without C10-15, Pam3, or Pam2 by replacing the culture medium. After 18 h of stimulation, HEK cells were gently washed once with 200 mL dPBS and then fixed with 4% paraformaldehyde in 150 mL dPBS for 10 min at RT. Cells were washed a second time and then stained with Hoechst 33342 (Invitrogen) at 1:10,000 dilution in dPBS for 8 min at RT. After the last wash, cells were mounted with Mounting Medium (Ibidi) and imaged using the Zeiss LSM 800 AiryScan Inverted Confocal Microscope at ×400 magnification.

### RT-qPCR analysis

Cultures of 2 × 10^6^ BMDMs were seeded into 12-well plates after 7 days of differentiation. After 1 day of resting, cells were stimulated with HDM, *C. albicans*, and TLR ligands for the indicated times. Cells were washed with dPBS and lysed in 350 mL RLT buffer (Qiagen) containing 1% (v/v) β-mercaptoethanol, and total RNA was isolated using the RNeasy Mini kit (Qiagen) following DNA digestion (RNase-free DNase set, Qiagen) and using a QIAcube instrument. The concentration of total RNA was measured using Nanodrop. mRNA reverse transcription to cDNA was performed using the High Capacity RNA-to-cDNA Kit (Thermo Fisher). Quantitative PCR was performed in a 10-mL mix containing 1 to 10 diluted cDNA, TaqMan Universal MasterMix II, and 0.3 mM TaqMan primer and RNA-free water. Each sample was analyzed in triplicates in a real-time cycler (Thermo QuantStudio 7 Flex, Thermo Fisher). The following cycle was used: 10 min/95°C; 15 s/95°C and 1 min/60°C for 40 cycles; and cooled and stored. The primers used are listed in **Supplementary Table S5**.

### Fitting of a chitin decamer into a TLR1-2 heterodimer structure

To visualize a chitin decamer bound to a TLR1/TLR2 heterodimer *in silico* (**Figure 4J**), docking and an energy minimization simulation were performed as described previously (95). Briefly, 3D structures of different GlcNAc oligomers created with the Glycam Carbohydrate Builder (GLYCAM Web, at legacy.glycam.org) were docked into the monomers of the TLR1/TLR2 heterodimer (PDB: 2Z7X; 38) using AutoDock Vina-Carb 1.1.2 (96). One docking state each of (GlcNAc)_8_ in TLR1 and (GlcNAc)_10_ in TLR2 were selected and combined via PyMOL (Schrödinger) by aligning and pair fitting to create a TLR1/TLR2 heterodimer with a bound chitin decamer. This new complex is based on the mentioned crystal structure but features a slightly higher distance between the receptor monomers to allow optimal accommodation of the decamer. The outcome was finally optimized using the GROMACS 2019 (97) package with the force fields Amber ff14SB (98) and GLYCAM06 (99) by running an energy minimization simulation with the preparation and setup described previously (95).

### Statistical analysis and software

Experimental data were analyzed in GraphPad Prism 8.3.1 (GraphPad Software, Inc.). Normal distribution was not formally tested but considered to apply judging from the data distribution. Hence, two-tailed Student’s *t*-tests and one-way or two-way ANOVA tests were used and adjusted for multiple testing as suggested by the analysis software and as indicated. Microscopy data were obtained using the ZEN blue (Zeiss) software and analyzed using ImageJ and Fiji. p-Values < 0.05 were generally considered statistically significant and were denoted by an asterisk throughout the figure legends, even if the actual p-values were considerably lower. Schematic images were created with BioRender.com.

## Data Availability

Requests to access the datasets should be directed to AW, alexander.weber@uni-tuebingen.de.

## Supplementary material

Supplementary Table 1 List of TLR agonists, recombinant proteins and inhibitors.

Supplementary Table 2 List of plasmids.

Supplementary Table 3 Mutagenesis primers to generate catalytically inactive mutant chitinases.

Supplementary Table 4 List of antibodies.

Supplementary Table 5 List of qPCR primers.
