# Supplementary figures and images for: Transkingdom mechanism of MAMP generation by chitotriosidase feeds oligomeric chitin from fungal pathogens and allergens into TLR2-mediated innate immune sensing
Source: Front Immunol. 2025 Mar 3;16:1497174. doi: 10.3389/fimmu.2025.1497174 (PMC11911531; doi:10.3389/fimmu.2025.1497174)

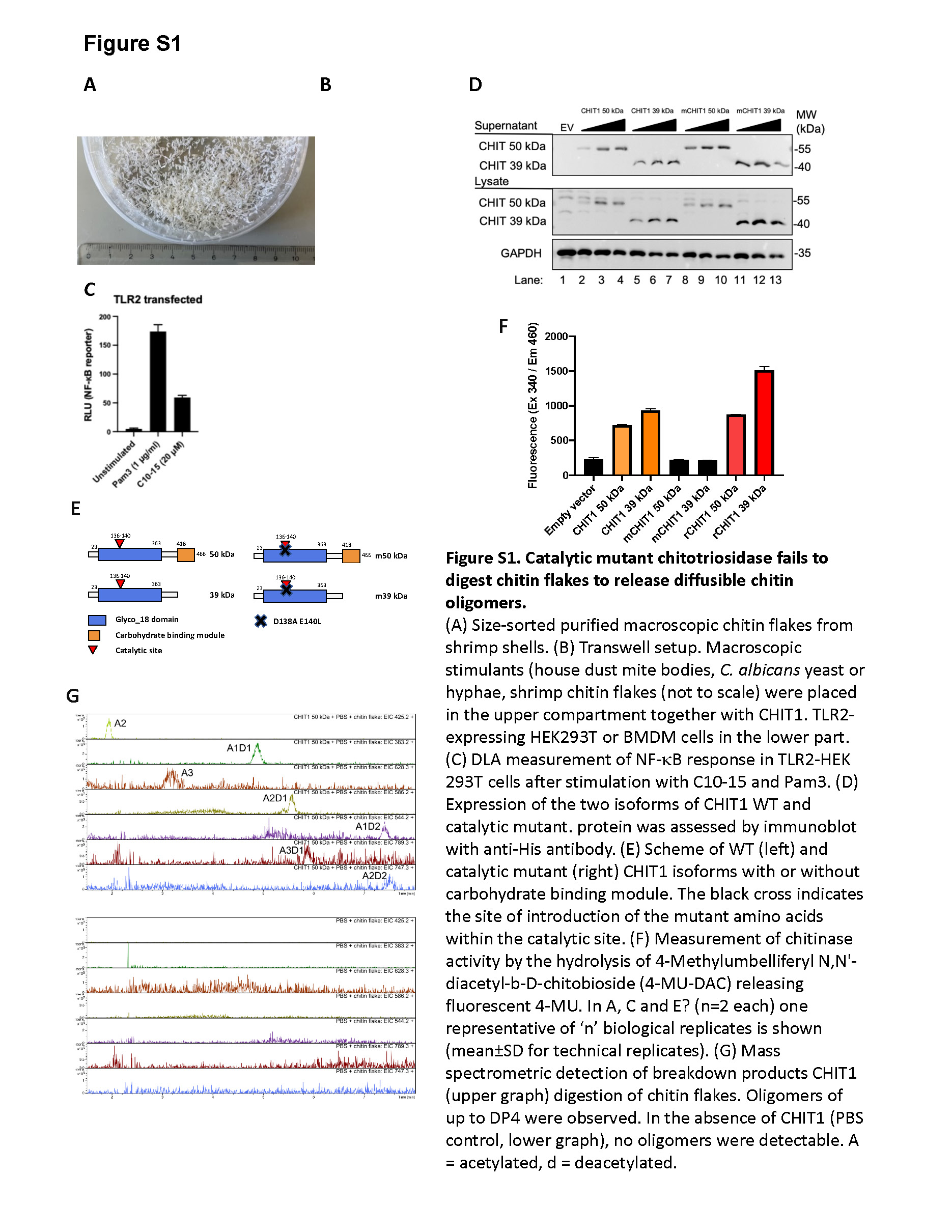

Supplement: Supplementary Table 1 — List of TLR agonists, recombinant proteins and inhibitors. [file Image1.jpeg]

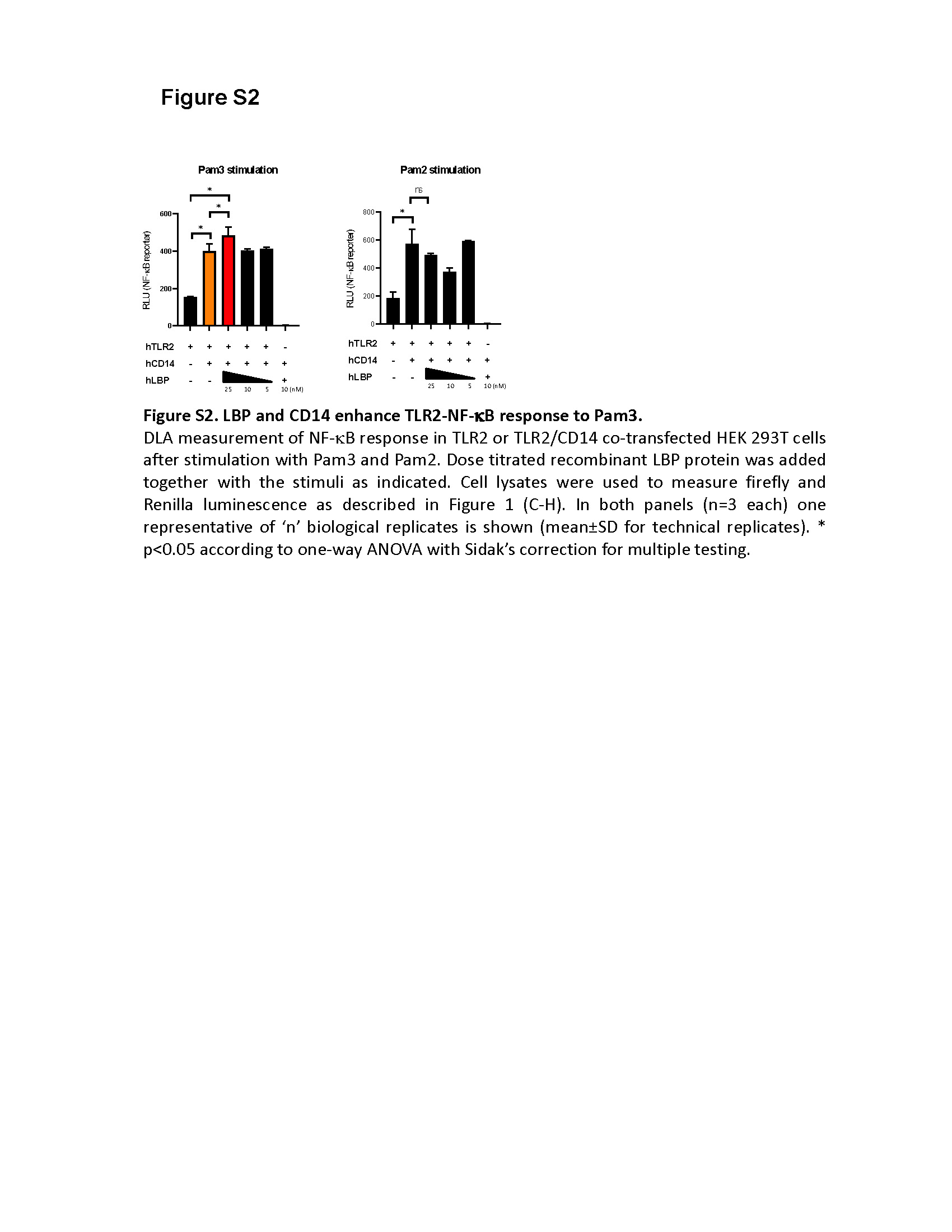

Supplement: Supplementary Table 2 — List of plasmids. [file Image2.jpeg]

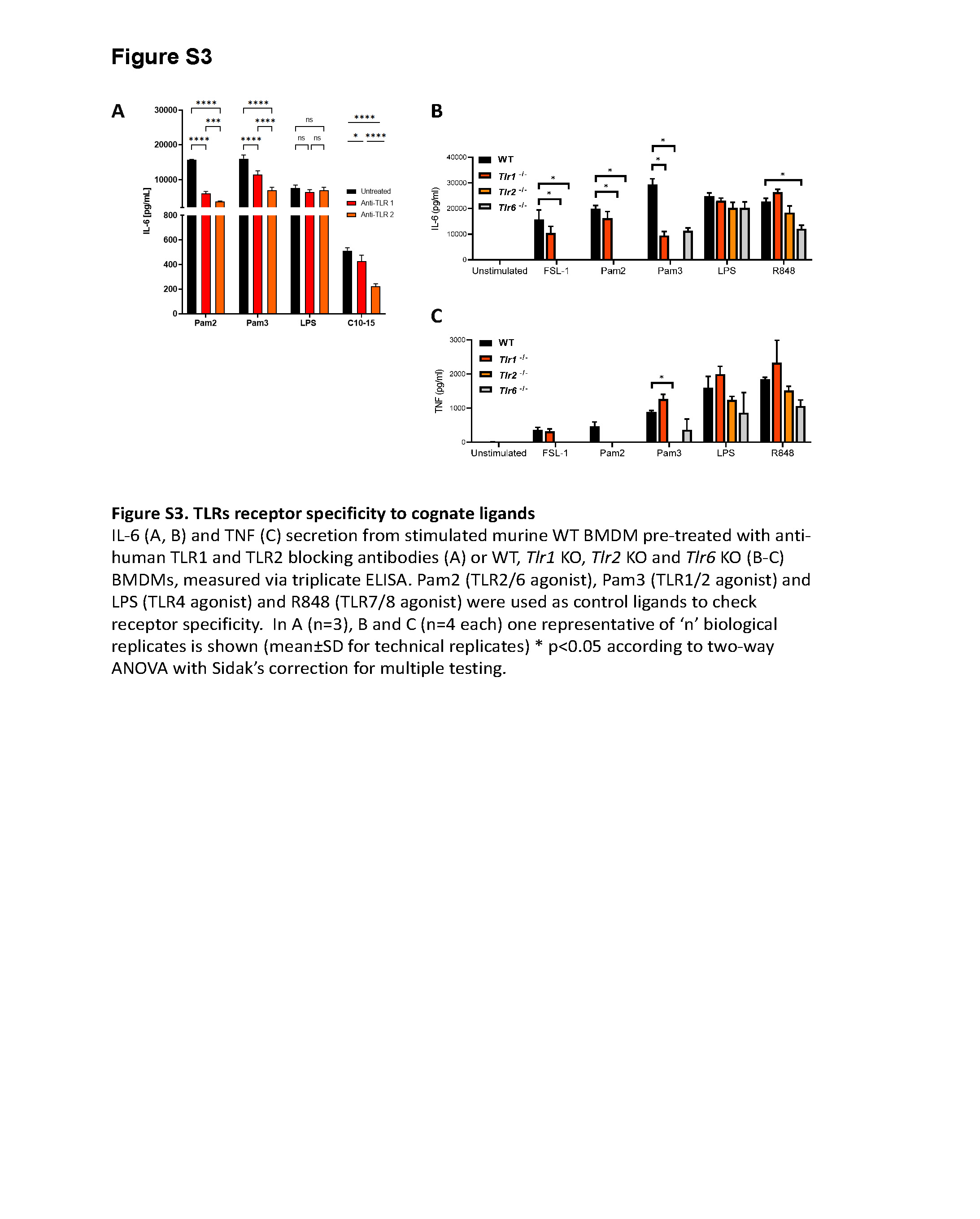

Supplement: Supplementary Table 3 — Mutagenesis primers to generate catalytically inactive mutant chitinases. [file Image3.jpeg]

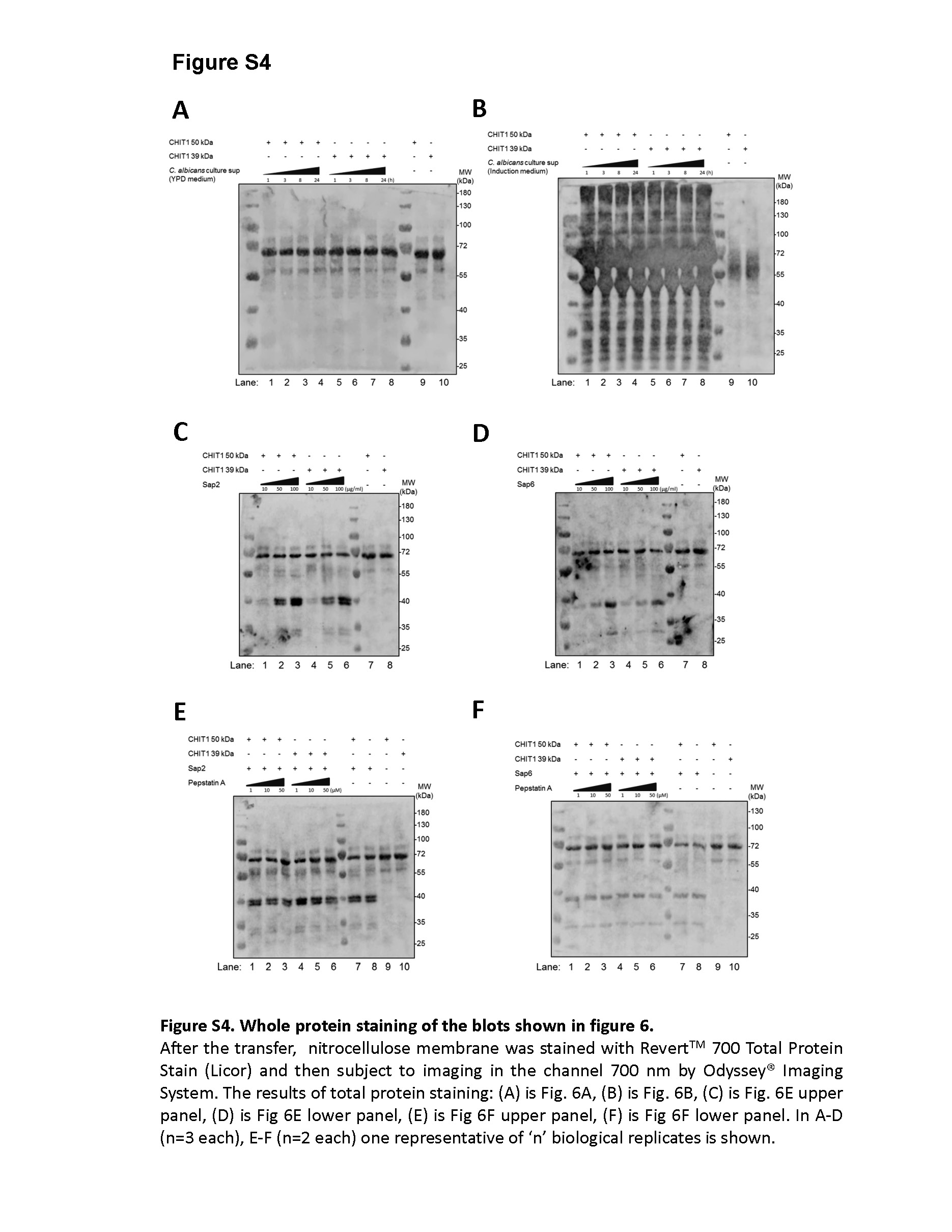

Supplement: Supplementary Table 4 — List of antibodies. [file Image4.jpeg]
