# Supplementary material for: Transkingdom mechanism of MAMP generation by chitotriosidase feeds oligomeric chitin from fungal pathogens and allergens into TLR2-mediated innate immune sensing
Source: Front Immunol. 2025 Mar 3;16:1497174. doi: 10.3389/fimmu.2025.1497174 (PMC11911531; doi:10.3389/fimmu.2025.1497174)
Supplement: Supplementary Table 5 — List of qPCR primers. [file Table1.docx]

# Supplemental information

**Supplemental tables**

Table S1. List of TLR agonists, recombinant proteins and inhibitors

| Ligands | Final concentration | Catalog ID | Origin |
| --- | --- | --- | --- |
| C10-15 (oligomeric chitin, generated from chitosan) | 20 mM | OC28900 | Carbosynth, see Methods |
| Zymosan | 100 mg/ml | tlrl-zyn | Invivogen |
| Pam3 | 40 nM to 1 mM | tlrl-pms | Invivogen |
| Pam2 | 40 nM to 1 mM | tlrl-pm2s-1 | Invivogen |
| FSL-1 | 40 nM to 1 mM | tlrl-fsl | Invivogen |
| LPS | 100 ng/ml | tlrl-eklps | Invivogen |
| R848 | 5 mg/ml | tlrl-r848-5 | Invivogen |
| Proteins | Concentration | Catalog ID | Origin |
| rCHIT1 50 kDa,  rCHIT1 39 kDa | 1 mM | - | This study, ([Stockinger et al., 2015](#_ENREF_81)) |
| Zymolase | 25 nM | tlrl-zyn | Invivogen |
| hLBP | 5-25 nM | ab151656 | Abcam |
| Sap2 and Sap6 | 10-100 µg/ml | - | ([Gropp et al., 2009](#_ENREF_30)) |
| Inhibitors | Concentration | Catalog ID | Origin |
| Polymyxin B | 10 mg/ml | 21850029 | Thermo Fisher |
| Caspofungin | 0.032 mg/ml | SML0425 | Sigma-Aldrich |
| Pepstatin A | 1-50 µM | 40311360025 | Bachem |

Table S2. List of plasmids

| **Insert and source if applicable** | **Tag** | **Backbone** | **Resistance** |
| --- | --- | --- | --- |
| Empty | n/a | pcDNA3 | Ampicillin |
| hTLR2 | Flag | pcDNA3 | Ampicillin |
| Empty (for BP reaction) | n/a | pDONR207 | Gentamycin |
| hTLR2 (open for gateway)  Harvard Plasmid repository | n/a | pDONR221 | Kanamycin |
| hTLR1 (open for gateway)  Harvard Plasmid repository | n/a. | pENTR223 | Spectinomycin |
| hCHIT1 50 kDa ([Stockinger et al., 2015](#_ENREF_81)) | His | pTT5V5H8Q | Ampicillin |
| hCHIT1 39 kDa ([Stockinger et al., 2015](#_ENREF_81)) | His | pTT5V5H8Q | Ampicillin |
| Empty  Gateway adapted plasmid ([Weiler et al., 2014](#_ENREF_94)) was a gift from Prof. Qingming Luo | Myc | pDEST_Myc-LC151 | Ampicillin |
| Empty  Gateway® adapted plasmid Ref. PMID: 24367102, original plasmid is a gift by Prof. Qingming Luo | HA | pDEST_HA-LN151 | Ampicillin |
| hTLR2-LC151 | Myc | pDEST_Myc-LC151 | Ampicillin |
| hTLR2-LN151 | HA | pDEST_HA-LN151 | Ampicillin |
| hTLR1-LC151 | Myc | pDEST_Myc-LC151 | Ampicillin |
| hTLR1-LN151 | HA | pDEST_HA-LN151 | Ampicillin |
| attB-hTLR6  Genewiz synthesis | N.A. | pUC57 | Kanamycin |
| attL-hTLR6 (open for gateway) | N.A. | pDONR207 | Gentamycin |
| hTLR6-LC151 | Myc | pDEST_Myc-LC151 | Ampicillin |
| hTLR6-LN151 | HA | pDEST_HA-LN151 | Ampicillin |
| hCHT1 (50kDa) D138AE140L | His | pTT5V5H8Q | Ampicillin |
| hCHT2 (39kDa) D138AE140L | His | pTT5V5H8Q | Ampicillin |
| NF-kB firefly luciferase reporter (Promega) | N.A. | pNF-kB | Ampicillin |
| *Renilla* luciferase (Promega) | N.A. | pRL-TK | Ampicillin |

Table S3. Mutagenesis primers to generate catalytically inactive mutant chitinases

| **Primer name** | **Sequence (5' to 3')** |
| --- | --- |
| CHIT1 D138AE140L Fwd | 5‘-CTTGACCTTGCCTGGCTGTACCCAGGAAGC -3’ |
| CHIT1 D138AE140L Rev | 5’- GCTTCCTGGGTACAGCCAGGCAAGGTCAAG -3’ |

Table S4. List of antibodies

| **Antibodies** | **Company** | **Specious** | **Catalog No.** | **Dilutions** |
| --- | --- | --- | --- | --- |
| Anti-GAPDH (GA1R) | Thermal Fisher | Mouse | MA5-15738 | 1:5000 |
| Anti-TLR1 polyclonal Ab | Cell Signaling | Rabbit | 2209 | 1:1000 |
| Anti-TLR2 (D7G9Z) mAb | Cell Signaling | Rabbit | 12276 | 1:1000 |
| Anti-TLR6 (D1Z8B) mAb | Cell Signaling | Rabbit | 362609 | 1:1000 |
| Anti-human TLR1 (H2G2) blocking Ab | Invivogen | mouse | mabg-htlr1-2 | 1:25 |
| Anti-human TLR2 (T2.5) blocking Ab | Invivogen | mouse | mab2-mtlr2 | 1:25 |
| Anti-Penta His | Qiagen | Mouse | 34660 | 1:2000 |
| Anti-Mouse HRP conjugate | Promega | Goat | W4028 | 1:10000 |
| Anti-Rabbit HRP conjugate | Vector | Goat | PI-1000 | 1:5000 |
| Anti-His Alexa Fluor® 594 conjugated | Biolegend | Mouse | 362609 | 1:100 |

Table S5. List of qPCR primers

| **Gene** | **Primer number** | **Catalog number** |
| --- | --- | --- |
| *mTbp* | Mm00446971_m1 | [4331182](https://www.thermofisher.com/order/catalog/product/4331182) |
| *mIl6* | Mm00446190_m1 | 4331182 |
| *mChit1* | Mm01291360_m1 | [4331182](https://www.thermofisher.com/order/catalog/product/4331182) |
